# Ribonuclease 1 Induces T‐Cell Dysfunction and Impairs CD8^+^ T‐Cell Cytotoxicity to Benefit Tumor Growth through Hijacking STAT1

**DOI:** 10.1002/advs.202404961

**Published:** 2025-02-11

**Authors:** Wen‐Hao Yang, Bao‐Yue Huang, Hsing‐Yu Rao, Peng Ye, Bi Chen, Hao‐Ching Wang, Chih‐Hung Chung, Heng‐Hsiung Wu, Hung‐Rong Yen, Shao‐Chun Wang, Jong‐Ho Cha, Xiuwen Yan, Muh‐Hwa Yang, Mien‐Chie Hung

**Affiliations:** ^1^ Graduate Institute of Cell Biology and Cancer Biology and Precision Therapeutics Center China Medical University Taichung 406040 Taiwan; ^2^ Affiliated Cancer Hospital and Institute of Guangzhou Medical University Guangzhou Guangdong 910095 China; ^3^ Infection Medicine Research Institute of Panyu District The Affiliated Panyu Central Hospital of Guangzhou Medical University Guangzhou Guangdong 910095 China; ^4^ The PhD Program for Translational Medicine, and Graduate Institute of Translational Medicine College of Medical Science and Technology Taipei Medical University Taipei 110301 Taiwan; ^5^ Cancer and Immunology Research Center National Yang Ming Chiao Tung University Taipei 112304 Taiwan; ^6^ Graduate Institute of Biomedical Sciences China Medical University Taichung 404328 Taiwan; ^7^ School of Chinese Medicine College of Chinese Medicine China Medical University Taichung 404328 Taiwan; ^8^ Department of Biomedical Science and Engineering Graduate School Inha University Incheon 22212 Republic of Korea; ^9^ Institute of Clinical Medicine and Cancer and Immunology Research Center National Yang Ming Chiao Tung University Taipei 112304 Taiwan; ^10^ Department of Oncology Taipei Veterans General Hospital Taipei 112201 Taiwan; ^11^ Graduate Institute of Biomedical Sciences Institute of Biochemistry and Molecular Biology Research Center for Cancer Biology Cancer Biology and Precision Therapeutics Center and Center for Molecular Medicine China Medical University Taichung 406040 Taiwan

**Keywords:** effector cytokine, immune checkpoint protein, ribonuclease 1, STAT1, T‐cell dysfunction

## Abstract

T‐cell‐based immunotherapy holds promise for eliminating cancer through T‐cell activation. However, prolonged interaction between T cells and tumors and the presence of immunosuppressive factors can diminish T‐cell cytotoxicity, leading to treatment failure. Here, ribonuclease 1 (RNase1), which degrades RNA, reduced the expression of effector cytokines and increases immune checkpoint protein levels, inducing T‐cell dysfunction. RNase1 expression is positively associated with exhausted T‐cell gene signatures and immune checkpoint proteins across several cancer types. Cancer cells expressing RNase1 are resistant to CD8^+^ T‐cell‐mediated killing. RNase1 promotes tumor growth in immunocompetent, but not in immunodeficient, mouse models and inhibits CD8^+^ T‐cell activity in vivo. Mechanistically, RNase1 enters T cells and deactivates signal transducer and activator of transcription 1 (STAT1), causing T‐cell dysfunction. Loss of RNase1‐STAT1 interaction restores CD8^+^ T‐cell cytotoxicity. Notably, a study has found RNase1 might activate CD4^+^ T cells to inhibit breast cancer growth, while another has indicated it causes immunosuppression in liver cancer. The current research shows that RNase1 does not impact CD4^+^ T cells in vivo. Overall, the study supports the immunosuppressive role of RNase1 in cancer of negatively regulating STAT1 to impair CD8^+^ T‐cell cytotoxicity. Targeting the RNase1‐STAT1 interaction could prevent CD8^+^ T‐cell dysfunction in RNase1‐highly expressing cancer patients.

## Graphical abstract

T‐cell immunotherapy eliminates cancer growth by activating T cells, but immunosuppressive factors hinders their effectiveness, resulting in treatment failure. Here, ribonuclease 1 (RNase1) causes T‐cell dysfunction and promotes exhausted T‐cell signatures in cancers. RNase1 enables cancer cells to prevent CD8+ T‐cell attacks through signal transducer and activator of transcription 1 (STAT1) inactivation, causing T‐cell dysfunction and underscoring RNase1's immunosuppressive role.

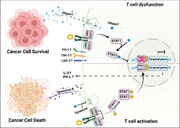

## 1 Introduction

The interaction between programmed death‐ligand 1 (PD‐L1) on cancer cells and programmed cell death protein 1 (PD‐1) on activated T cells is a well‐established inhibitory signaling of immune checkpoint (IC) regulation, which suppresses effector T‐cell function and promotes cancer progression.^[^ ^1^ ^]^ Consequently, immune checkpoint inhibitors (ICIs), particularly antibodies targeting PD‐1 or PD‐L1, have demonstrated sustained clinical efficacy in individuals with advanced malignancies because they reinvigorate T cells to eliminate malignant cells.^[^ ^2^ ^]^ Furthermore, the use of adoptive cellular therapies, such as chimeric antigen receptor T‐cell therapy and T‐lymphocyte (T‐cell)‐based cancer immunotherapies, has revealed that activated T cells possess an enhanced ability to eradicate cancer cells.^[^ ^3^ ^]^ Nevertheless, a restricted subset of patients exhibits an initial response to these cancer immunotherapies, while a majority experience disease relapse and progression despite the presence of T cells within the tumor, even after an initially positive treatment response,^[^ ^4^ ^]^ suggesting that specific factors within the tumor microenvironment (TME) may induce T‐cell dysfunction and thereby contribute to immunotherapy failure. Furthermore, tumor‐infiltrating T cells may experience exhaustion, leading to a decline in their cytotoxic activity against cancer cells within the TME. This exhaustion is characterized by reduced effector cytokines and cytolytic molecules, coupled with upregulation of inhibitory immune checkpoint proteins such as lymphocyte activation gene 3 (LAG‐3), T‐cell immunoglobulin and mucin domain 3 (TIM‐3), and PD‐1, as well as their interaction with galectin‐9.^[^ ^5^ ^]^ Therefore, identifying barriers within the TME that impede T‐cell function is crucial to enhancing the effectiveness of T‐cell‐based cancer immunotherapies.

Proteins secreted by tumor cells or stromal cells within the TME may serve as serum biomarkers for tumor diagnosis, prognosis, and treatment response monitoring. These secreted proteins influence tumor progression and therapeutic outcomes through autocrine or paracrine signaling and mediate the crosstalk between tumor cells and stromal cells, including immune cells. Studies have reported that proteins secreted by cancer cells can promote cancer proliferation, stemness, and metastasis.^[^ ^6^ ^]^ In the context of cancer immunity, various secreted cytokines, including interferon alpha (IFN‐α), IFN‐γ, interleukin‐2 (IL‐2), and granulocyte macrophage‐colony stimulating factor (GM‐CSF), play crucial roles in mediating immune responses and have been employed in cancer immunotherapy. For example, the Food and Drug Administration has approved the use of IFN‐α and IL‐2 for anticancer treatments.^[^ ^7^ ^]^ Therefore, the cancer secretome influences the behavior of both neoplastic and non‐neoplastic cells, making them promising sources of biomarkers.^[^ ^8^ ^]^ Additionally, cancer‐secreted proteins have been reported to be both potential diagnostic indicators and targets for cancer immunotherapy.^[^ ^9^ ^]^ Further investigation into newly identified cancer cell‐secreted proteins, particularly those with immune checkpoint‐like properties that induce T‐cell dysfunction, may provide compelling avenues for developing serum marker‐guided immunotherapies aimed at restoring T‐cell function in the TME and enhancing the efficacy of T‐cell‐based immunotherapies in cancer treatment.

The human RNaseA (hRNaseA) superfamily comprises 13 members of secretory RNases, which can be secreted into the extracellular space and plasma or serum. They are categorized into two groups: the canonical subgroup (RNases 1–8) and the noncanonical subgroup (RNases 9–13). The noncanonical subgroup lacks enzymatic activity.^[^ ^10^ ^]^ These hRNaseA superfamily proteins are primarily known for their function of degrading RNA. In healthy individuals, RNases predominantly contribute to the immune response against bacterial or viral infections by degrading RNA or participate in the digestive system to facilitate nutrient absorption.^[^ ^10b^ ^]^ Their pathological roles in cancer have been gradually unveiled recently. For instances, ribonuclease 1 (RNase1), RNase4, RNase5, and RNase7 have been reported to function as ligands for cell membrane receptors, promoting cancer malignancy independently of their ribonucleolytic activity. Additionally, they may serve as potential biomarkers for tumorigenesis and progression,^[^ ^6^, ^11^ ^]^ indicating broader biological effects of the hRNaseA superfamily beyond RNA degradation. It has been known for a while that RNases can enter cells through endocytosis to degrade cellular RNA,^[^ ^12^ ^]^ thus it also raises an interesting question if RNases play roles independent of ribonucleolytic activity after endocytotic internalization.

Because studies have predominantly focused on membrane‐bound immune checkpoint proteins such as PD‐L1, PD‐L2, and B7‐H3 on tumor cells, which aid in evading immune surveillance, and T cells play a key role in these regulations of cancer immunity,^[^ ^13^ ^]^ the role of cancer cell‐secreted proteins in T‐cell dysfunction has received limited attention. Therefore, in this study, we investigated whether any cancer cell‐secreted proteins contribute to T‐cell dysfunction or inactivation. We employed an unbiased screening approach and unexpectedly discovered that secretory RNase1 can directly induce T‐cell dysfunction and promote features associated with T‐cell exhaustion. Our findings indicate that RNase1 enhances the characteristics of dysfunctional T cells, including those of diminished CD8^+^ T cell cytotoxicity, elevated expression of immune inhibitory checkpoint proteins, and decreased levels of effector cytokines. Mechanistically, we identified a novel pathway whereby RNase1 internalizes into T cells and interacts with signal transducer and activator of transcription 1 (STAT1) to inhibit STAT1‐mediated CD8^+^ T‐cell cytotoxicity. These results indicate that RNase1 is a potential serum biomarker for predicting the efficacy of CD8^+^ T‐cell‐based immunotherapy. Moreover, targeting the RNase1‐STAT1 interaction presents a promising strategy for preventing T‐cell dysfunction.

## 2 Results

### 2.1 Cancer Cell‐Secreted RNase1 Contributes to T‐Cell Dysfunction and Promotes Exhausted T Cell Features

**Figure 1:**
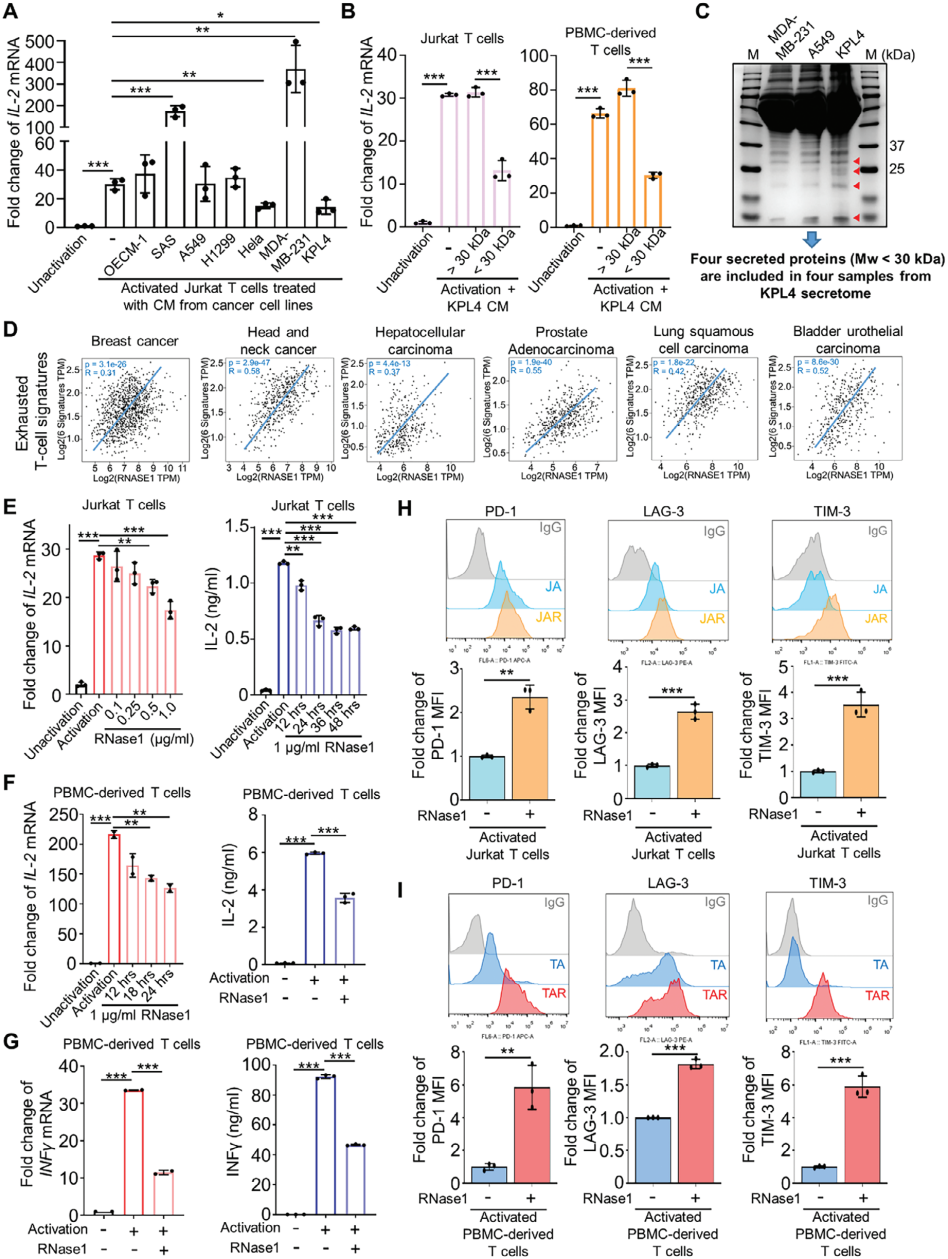
RNase1 is a candidate for inducing T‐cell dysfunction in cancer cell‐secreted proteins to promote exhausted T cell features. A) Quantitative RT‐PCR analysis of *IL‐2* mRNA expression in unactivated‐Jurkat T cells and activated Jurkat T cells treated without or with CM from different cancer cell lines for 48 h. Representative data from three independent experiments (each experiment contains three technical replicates). B) Left: Quantitative RT‐PCR analysis of *IL‐2* mRNA expression in unactivated Jurkat T cells and activated Jurkat T cells treated without or with the two fractions (molecular weight larger than 30 kDa and smaller than 30 kDa) of KPL4 CM for 48 h. Right: Quantitative RT‐PCR analysis of *IL‐2* mRNA expression in unactivated peripheral blood mononuclear cells (PBMC)‐derived T cells and activated PBMC‐derived T cells treated without or with the two fractions (molecular weight larger than 30 kDa and smaller than 30 kDa) of KPL4 CM for 48 h. Representative data from three independent experiments (each experiment contains three technical replicates). C) The increased proteins (indicated by red arrows) in CM of KPL4 cells compared to that of MDA‐MB‐231 and A549 cells separated by SDS‐PAGE and identified by mass spectrum analysis. D) Correlation analysis between RNase1 expression and T cell exhaustion signatures in patient tissues of breast cancer, HNSC, HCC, prostate adenocarcinoma, lung squamous cancer, and bladder urothelial carcinoma from the GEPIA2 database. E) Left: Quantitative RT‐PCR analysis of *IL‐2* mRNA expression in unactivated Jurkat T cells and activated Jurkat T cells treated without or with different concentrations of recombinant RNase1 as indicated for 48 h. Right: CM collected from unactivated Jurkat T cells and activated Jurkat T cells treated without or with 1 µg mL^−1^ recombinant RNase1 for different time‐points as indicated were subjected to enzyme‐linked immunosorbent assay (ELISA) assay to determine IL‐2 concentrations. Representative data from three independent experiments (each experiment contains three technical replicates). F) Left: Quantitative RT‐PCR analysis of *IL‐2* mRNA expression in unactivated PBMC‐derived T cells and activated PBMC‐derived T cells treated without or with 1 µg mL^−1^ recombinant RNase1 for different time‐points as indicated. Representative data from three independent experiments (each experiment contains two technical replicates). Right: CM collected from unactivated PBMC‐derived T cells and activated PBMC‐derived T cells treated without or with 1 µg mL^−1^ recombinant RNase1 for 48 h were subjected to ELISA assay to determine IL‐2 concentrations. Representative data from three independent experiments (each experiment contains three technical replicates). G) Left: Quantitative RT‐PCR analysis of INF‐γ mRNA expression in unactivated PBMC‐derived T cells and activated PBMC‐derived T cells treated without or with 1 µg mL^−1^ recombinant RNase1 for 48 h. Representative data from three independent experiments (each experiment contains two technical replicates). Right: CM collected from unactivated PBMC‐derived T cells and activated PBMC‐derived T cells treated without or with 1 µg mL^−1^ recombinant RNase1 for 48 h were subjected to ELISA assay to determine INF‐γ concentrations. Representative data from three independent experiments (each experiment contains three technical replicates). H) Membrane PD‐1, LAG‐3, and TIM‐3 expression by flow cytometric analysis after activated Jurkat T cells were treated with 1 µg mL^−1^ recombinant RNase1 for 48 h compared with untreated cells. Upper panel: Representative data of flow cytometric analysis. Lower panel: Quantitative data of flow cytometric analysis from three independent experiments. I) Membrane PD‐1, LAG‐3, and TIM‐3 expression by flow cytometric analysis after activated PBMC‐derived T cells were treated with 1 µg mL^−1^ recombinant RNase1 for 48 h compared with untreated cells. Upper panel: Representative data of flow cytometric analysis. Lower panel: Quantitative data of flow cytometric analysis from three independent experiments. Data represent mean ± SD, *p, 0.01–0.05, **p, 0.001–0.01, ***p < 0.001 by two‐sided unpaired Student's *t*‐test.

To identify cancer cell‐secreted proteins that inhibit T‐cell activity, we treated activated Jurkat T cells with the conditioned medium (CM) obtained from various cancer cell lines. Because IL‐2 is a key cytokine and marker of T‐cell activation,^[^ ^14^ ^]^ we assessed the messenger ribonucleic acid (mRNA) levels of IL‐2 in these untreated and CM‐treated groups of activated Jurkat T cells. Notably, treatment with CM derived from breast and cervical cancer cell lines, that is, KPL4 and Hela cells, led to a significant reduction in IL‐2 mRNA expression in activated Jurkat T cells compared with that in the untreated control (**Figure** **1**A). This observation indicates that some secretory proteins from KPL4 or Hela cells may impede T‐cell activation. To elucidate which components of the CM from cancer cells might be responsible for suppressing IL‐2 expression in activated T cells, we fractionated the CM obtained from KPL4 on the basis of molecular weight (*M*_w_) and separated it into fractions larger than 30 kDa and smaller than 30 kDa by using centrifugal filters. Only the smaller fraction of KPL4 CM inhibited IL‐2 mRNA expression in both activated Jurkat T cells and T cells derived from human peripheral blood mononuclear cells (PBMCs; Figure 1B). Compared to CM from MD Anderson‐Metastatic Breast‐231 (MDA‐MB‐231) and A549 cells, which cannot decrease IL‐2 mRNA expression of activated‐Jurkat T cells (Figure 1A), four protein bands with an *M*_w_ of < 30 kDa exhibited higher expression levels in KPL4 CM (Figure 1C, red arrows). Mass spectrometric (MS) analysis of these four protein bands revealed that four secreted proteins with an *M*_w_ less than 30 kDa, including Tetranectin isoform 2 (TN, *M*_w_ 17.7 kDa), hemoglobin subunit alpha (HBA1, *M*_w_ 15.1 kDa), RNase1 (*M*_w_ 17.6 kDa), and retinol‐binding protein 4 isoform b (RBP4, *M*_w_ 22.8 kDa), appeared in all samples of MS analysis (Figure 1C). While searching for potential T cell functions of these four candidates in the literature, RNase1 was the one that stands out as the most promising candidate.^[^ ^11^, ^15^ ^]^ Therefore, we analyzed the Cancer Genome Atlas (TCGA) database and discovered a significant positive correlation between RNase1 expression and exhausted T‐cell signatures across six cancer types (Figure 1D). We used quantitative real‐time reverse‐transcription polymerase chain reaction and enzyme‐linked immunosorbent assay (ELISA) assays to respectively assess IL‐2 mRNA expression in activated Jurkat T cells and IL‐2 concentrations in CM following recombinant RNase1 treatment, and we observed a dose‐dependent repression of IL‐2 mRNA levels and a time‐dependent decrease in IL‐2 concentrations in CM from activated Jurkat T cells (Figure 1E). Similarly, T cells derived from PBMCs treated with RNase1 also exhibited reduced IL‐2 mRNA levels and protein concentrations in CM (Figure 1F). Because dysfunctional or exhausted T cells typically exhibit diminished production of effector cytokines,^[^ ^16^ ^]^ we examined the impact of RNase1 treatment on other effector cytokines in activated T cells. Our results revealed that RNase1 treatment led to reductions in INF‐γ release and mRNA levels of INF‐γ (Figure 1G), IL‐6 (Figure S1A, Supporting Information), and GM‐CSF (Figure S1B, Supporting Information) in activated T cells. The critical character of T‐cell exhaustion is the increase of multiple ICs, such as PD‐1, LAG‐3, and TIM‐3. Our results of flow cytometry analysis revealed that RNase1 treatment significantly upregulated the expression of PD‐1, LAG‐3, and TIM‐3 on both activated Jurkat and PBMC‐derived T cells (Figure 1H,I). These findings collectively suggest that RNase1 can induce T‐cell dysfunction and exhausted features in vitro, which may contribute to its immunosuppressive role in the TME.

### 2.2 RNase1 Leads to the Dysfunction of CD4^+^ and CD8^+^ T Cells and Decreases the Cytotoxicity of CD8^+^ T Cells In Vitro, Independent of Its Ribonucleolytic Activity

**Figure 2:**
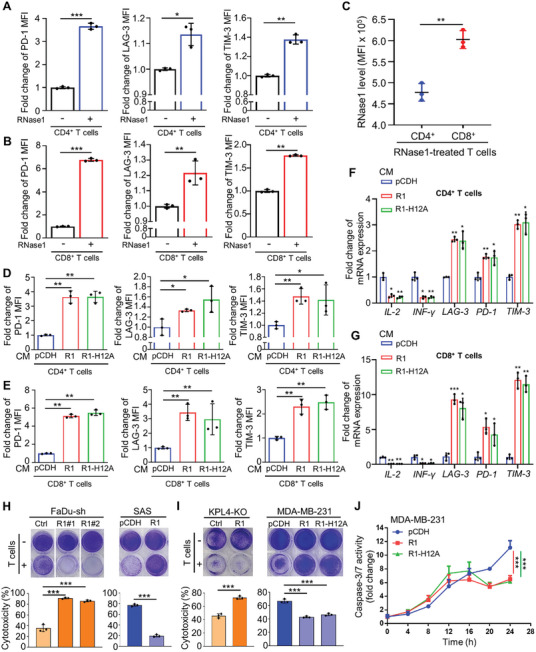
The ribonucleolytic activity‐independent function of RNase1 induces the dysfunction of CD4^+^ and CD8^+^ T cells, and reduces CD8^+^ T cell cytotoxicity in vitro. A) Membrane PD‐1, LAG‐3, and TIM‐3 expression by flow cytometric analysis in CD4^+^ T cells isolated from activated PBMC‐derived T cells treated with or without 1 µg mL^−1^ recombinant RNase1 for 24 h. Quantitative data of flow cytometric analysis from three independent experiments. B) Membrane PD‐1, LAG‐3, and TIM‐3 expression by flow cytometric analysis in CD8^+^ T cells isolated from activated PBMC‐derived T cells treated with or without 1 µg mL^−1^ recombinant RNase1 for 24 h. Quantitative data of flow cytometric analysis from three independent experiments. C) RNase1 levels detected by flow cytometric analysis in CD4^+^ and CD8^+^ T cells isolated from activated PBMC‐derived T cells treated with 1 µg mL^−1^ recombinant RNase1 for 24 h. Quantitative data of flow cytometric analysis from three independent experiments. D) Membrane PD‐1, LAG‐3, and TIM‐3 expression by flow cytometric analysis in CD4^+^ T cells isolated from activated PBMC‐derived T cells treated with CM collected from RNase1‐expressing (R1), enzyme‐dead RNase1‐expressing (R1‐H12A), or control (pCDH) MDA‐MB‐231 cells for 24 h. Quantitative data of flow cytometric analysis from three independent experiments. E) Membrane PD‐1, LAG‐3, and TIM‐3 expression by flow cytometric analysis in CD8^+^ T cells isolated from activated PBMC‐derived T cells treated with CM collected from R1, R1‐H12A, or pCDH MDA‐MB‐231 cells for 24 h. Quantitative data of flow cytometric analysis from three independent experiments. F) Quantitative RT‐PCR analysis of *IL‐2*, *INF‐γ*, *PD‐1*, *LAG‐3*, and *TIM‐3* mRNA expression in CD4^+^ T cells isolated from activated PBMC‐derived T cells treated with CM collected from R1, R1‐H12A, or pCDH MDA‐MB‐231 cells for 24 h. Representative data from three independent experiments (each experiment contains three technical replicates). G) Quantitative RT‐PCR analysis of *IL‐2*, *INF‐γ*, *PD‐1*, *LAG‐3*, and *TIM‐3* mRNA expression in CD8^+^ T cells isolated from activated PBMC‐derived T cells treated with CM collected from R1, R1‐H12A, or pCDH MDA‐MB‐231 cells for 24 h. Representative data from three independent experiments (each experiment contains three technical replicates). H) Representative images and quantitative results of T cell‐mediated cancer cell killing assay. Left: RNase1‐knockdown (sh‐R1#1 and #2) and control (sh‐Ctrl) FaDu cells (6 × 10^4^ cells) cocultured with or without activated PBMC‐derived T cells for 72 h were subjected to crystal violet staining to evaluate T‐cell cytotoxicity. FaDu to T‐cell ratio, 1:4. Right: RNase1‐expressing (R1), and control (pCDH) SAS cells (5 × 10^4^ cells) cocultured with activated PBMC‐derived T cells for 48 h were subjected to crystal violet staining to evaluate T‐cell cytotoxicity. SAS to T‐cell ratio, 1:6. Three independent experiments with three technical replicates were carried out. I) Representative images and quantitative results of T cell‐mediated cancer cell killing assay. Left: RNase1‐knockout (KO‐R1) and control (KO‐Ctrl) KPL4 cells (5 × 10^4^ cells) cocultured with or without activated PBMC‐derived T cells for 24 h were subjected to crystal violet staining to evaluate T‐cell cytotoxicity. KPL4 to T‐cell ratio, 1:4. Right: R1, R1‐H12A, and pCDH MDA‐MB‐231 cells (3 × 10^4^ cells) cocultured with or without activated PBMC‐derived T cells for 48 h were subjected to crystal violet staining to evaluate T‐cell cytotoxicity. MDA‐MB‐231 to T‐cell ratio, 1:4. Three independent experiments with three technical replicates were carried out. J) Time‐course quantitative results of T cell‐meditated cancer cell killing assay of dead cells. R1, R1‐H12A, and pCDH MDA‐MB‐231 cells (2000 cells) labeled with Incucyte Nuclight Rapid Red Dye were cocultured with CD8^+^ T cells isolated from activated PBMC‐derived T cells for 24 h. The caspase‐3/7 activity of dead cells was normalized to that at the zero‐time point. MDA‐MB‐231 to T‐cell ratio, 1:5. Two independent experiments with three technical replicates were carried out. Data are presented as mean ± SD, **p, 0.001–0.01, ***p < 0.001, and ns, not significant by A–I) two‐sided unpaired Student's *t*‐test or J) an ANOVA test.

To evaluate the impact of RNase1‐induced T‐cell dysfunction in T cell subsets, we assessed membrane PD‐1, LAG‐3, and TIM‐3 expression by flow cytometric analysis in CD4^+^ or CD8^+^ T cells isolated from activated PBMC‐derived T cells treated with or without RNase1. The results indicated that RNase1 treatment significantly increased membrane PD‐1, LAG‐3, and TIM‐3 expression in both CD4^+^ (**Figure** **2**A) and CD8^+^ T cells (Figure 2B). Interestingly, we also observed that CD8^+^ T cells might uptake more RNase1 than CD4 T cells (Figure 2C), suggesting CD8^+^ T cells may be more affected by RNase1‐induced dysregulation than CD4^+^ T cells. In the above results of flow cytometric analysis, the percentage of live T cells upon RNase1 treatment was not decreased (Figure S2A, Supporting Information). Since the enzyme activity of RNase1 may cause cell death, we further investigated whether 1 µg mL^−1^ of RNase1 treatment for 48 h affects the proliferation, apoptosis, and cell cycle of CD4^+^ and CD8^+^ T cells. The data showed that RNase1 had no significant effects on the proliferation (Figure S2B, Supporting Information), apoptosis (Figure S2C, Supporting Information), and cell cycle (Figure S2D, Supporting Information) of CD4^+^ and CD8^+^ T cells. In addition, the amount of total RNA in CD4^+^ and CD8+ T cells did not change after RNase1 treatment (Figure S2E, Supporting Information), suggesting that the ribonucleolytic activity of RNase1 in CD4^+^ and CD8^+^ T cells treated with 1 µg mL^−1^ RNase1 may be repressed or blocked.

Next, to define whether the dysfunction of CD4^+^ and CD8^+^ T cells by RNase1 requires its ribonucleolytic activity, we expressed RNase1 (R1) and a catalytically inactive RNase1 mutant (R1‐H12A)^[^ ^6b^ ^]^ in the low‐endogenous RNase1 expressing MDA‐MB‐231 breast cancer cells (Figure S2F, Supporting Information). The results of flow cytometric analysis indicated that both CD4^+^ and CD8^+^ T cells treated with CM obtained from MDA‐MB‐231‐R1 and R1‐H12A cells significantly increased membrane PD‐1, LAG‐3, and TIM‐3 expression compared to CM from MDA‐MB‐231‐pCDH (control) cells (Figure 2D,E). We also detected the decreases of mRNA expression of effector cytokines (IL‐2 and INF‐γ) and the increases of mRNA level of ICs (LAG‐3, PD‐1, and TIM‐3) in CD4^+^ and CD8^+^ T cells treated with CM from MDA‐MB‐231‐R1 and R1‐H12A cells compared with the control group (Figure 2F,G). These data support that RNase1‐induced CD4^+^ and CD8^+^ T‐cell dysfunction does not require the ribonuclease enzymatic activity in vitro.

**Figure 3:**
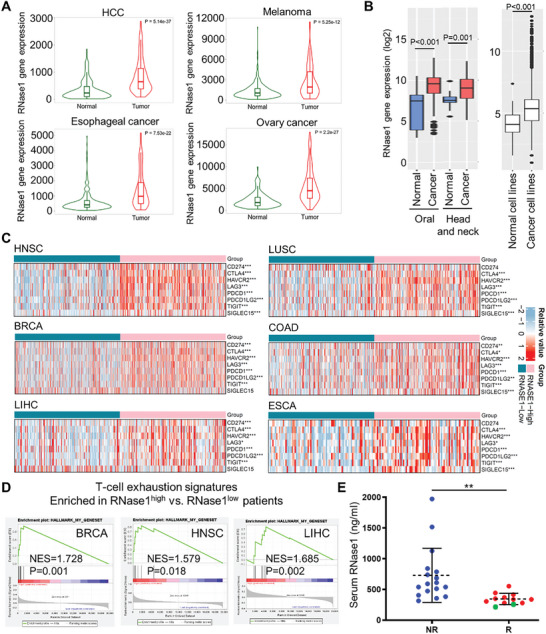
T cell exhaustion signatures are enriched in high RNase1‐expressing cancer patients and serum RNase1 predicts the poor efficacy of immune checkpoint inhibitors in clinics. A) RNase1 gene expression in patient tissues (T) of HCC, melanoma, esophageal cancer, and ovary cancer breast compared with noncancerous (N) individuals, assessed using the TNMplot analysis. B) RNase1 gene expression in oral cancer, head and neck cancer, or pan‐cancer cell lines compared with their normal control groups, assessed using GENT2 database. C) The heatmap of immune‐checkpoint‐related gene expression in cancer patient tissues of head and neck squamous cell carcinoma (HNSC), breast invasive carcinoma (BRCA), liver hepatocellular carcinoma (LIHC), lung squamous cell carcinoma (LUSC), colon adenocarcinoma (COAD), and esophageal carcinoma (ESCA) from the TCGA dataset. The patient tissues were separated into the RNase1‐low (green color) and high (pink color) groups. The different colors represent the trend of gene expression in different samples. The statistical difference of two groups was compared through the Wilcox test. *p < 0.05, **p < 0.01, ***p < 0.001. D) The gene set enrichment analysis (GSEA) results revealing enrichment of the T cell exhaustion signatures in the group with high RNase1 expression levels in the TCGA‐BRCA (*P* = 0.001, NES = 1.728), TCGA‐HNSC (*P* = 0.018, NES = 1.579) and TCGA‐LIHC (*P* = 0.002, NES = 1.685). E) Sera samples of HNSC patients from responders (R; *n* = 12) and nonresponders (NR; *n* = 17) to immune checkpoint inhibitor treatment were subjected to ELISA analysis with three technical replicates to determine RNase1 concentrations. Data are presented as mean ± SD, **p, 0.001–0.01 by two‐sided unpaired Student's *t*‐test.

Because dysfunctional T cells exhibit reduced cytotoxicity^[^ ^16b^ ^]^ and RNase1‐high expressing patients with head and neck squamous cell carcinoma (HNSC) and breast cancer associates with T‐cell exhaustion signatures (Figure 1D), we conducted T‐cell killing assays through RNase1 knockdown (sh‐R1#1 and sh‐R1#2) in FaDu HNSC cells or RNase1 knockout (KO‐R1) in KPL4 cells (Figure S2G,I, Supporting Information). Silencing or knocking out RNase1 in FaDu and KPL4 cells resulted in a significant increase in T cell‐mediated cancer cell killing compared to that in the control cells (Figure 2H,I; left panels). Additionally, we expressed RNase1 (R1) in the low‐endogenous RNase1 expressing cells, SAS human oral squamous cell carcinoma cells or MDA‐MB‐231 breast cancer cells (Figure S2F,H, Supporting Information) and discovered that RNase1 or catalytically inactive RNase1 mutant (R1‐H12A) expression reduced T‐cell cytotoxicity against cancer cells (**Figure** **3**H,I; right panels). The results indicated that even RNase1 lacking enzymatic activity still suppressed T cell‐mediated cancer cell killing (Figure 2I). To determinate the role of T cell subsets in T‐cell cytotoxicity repressed by RNase1, we first tested which T cell subsets were responsible for the primary T‐cell cytotoxicity against cancer cells. The results indicated that CD8^+^ T cells displayed the highest cytotoxicity, whereas CD4^+^ T cells showed very low cytotoxicity against MDA‐MB‐231 cancer cells in the in vitro T cell‐mediated cancer cell killing assay (Figure S2J, Supporting Information). Therefore, we further assessed the inhibition of T‐cell cytotoxicity by RNase1 in CD8^+^ T cells using a time‐course measurement of T cell‐mediated cancer cell killing effect. CD8^+^ T cells isolated from activated PBMC‐derived T cells were cocultured with MDA‐MB‐231‐pCDH, MDA‐MB‐231‐R1, or MDA‐MB‐231‐R1‐H12A cells labeled by an Incucyte Nuclight rapid red dye in the presence of an Incucyte caspase‐3/7 green dye combined with or without RNase1 treatment, and the green fluorescent caspase‐3/7 activity was measured to indicate apoptotic cell dead. The data showed that CD8^+^ T cell cytotoxicity began to be repressed after 16 h of coculture with MDA‐MB‐231‐R1 or MDA‐MB‐231‐R1‐H12A cells (Figure 2J). We also check the impacts of apoptosis, cell cycle, and total RNA amount in MDA‐MB‐231 cells treated with RNase1 and found that RNase1 treatment did not alter the cell percentage of apoptotic cells, cell phase distribution, and total RNA amount of MDA‐MB‐231 cells (Figure S2K–M, Supporting Information). Collectively, these data suggest that RNase1 can impede CD8^+^ T‐cell cytotoxicity in vitro, which does not require its ribonuclease enzymatic activity.

### 2.3 RNase1 Is Associated with T‐Cell Exhaustion Characteristics and Poor Efficacy of Immune Checkpoint Inhibitors in Samples from Cancer Patients

To verify whether RNase1‐induced T‐cell dysfunction possesses clinical implications and potential applications, we analyzed cancer patient tissues from the online databases. Our earlier studies have shown that RNase1 expression is higher in breast and liver cancer tissues compared to normal tissues.^[^ ^6^, ^11^ ^]^ Through a differential gene expression analysis in Tumor, Normal, and Metastatic tissues (TNM plot analysis),^[^ ^17^ ^]^ we revealed that hepatocellular carcinoma (HCC), melanoma, esophageal cancer, and ovarian cancer tissues express higher levels of RNase1 than normal tissues (Figure 3A). Moreover, an analysis of the GENT2 database^[^ ^18^ ^]^ revealed that RNase1 expression is not only elevated in head and neck cancer and oral cancer but also in cancer cell lines across various types compared with that in normal tissues and cell lines (Figure 3B). To further validate the association between RNase1 and ICs, we analyzed RNA‐sequencing expression profiles from the TCGA dataset. High levels of RNase1 in tissues from patients with cancer were significantly and positively correlated with the expression of ICs, including CD274 (PD‐L1), CTLA4, HAVCR2 (TIM‐3), PDCD1 (PD‐1), LAG‐3, PDCD1LG2 (PD‐L2), and T cell immunoreceptor with immunoglobulin and ITIM domain (TIGIT), in various cancers, such as HNSC, breast invasive carcinoma (BRCA), liver hepatocellular carcinoma (LIHC), lung squamous cell carcinoma (LUSC), colon adenocarcinoma (COAD), and esophageal carcinoma (ESCA) (Figure 3C). Further examination of these data by the gene set enrichment analysis (GSEA) revealed that T‐cell exhaustion signatures were enriched in the group of RNase1‐high expressing patients with BRCA, HNSC, and LIHC (Figure 3D). This finding provides additional support for the potential involvement of RNase1 in T‐cell dysfunction across various cancer types. We subsequently investigated the potent prognostic value of RNase1 as a serum biomarker for ICI treatment because T‐cell dysfunction in the TME has been associated with ICI treatment failure.^[^ ^4^, ^19^ ^]^ We collected serum samples from HNSC patients with recurrent or metastatic diseases undergoing ICI therapy and measured RNase1 concentrations by using ELISA assays. Compared with the responder group (R, *n* = 12) of HNSC patients, who exhibited positive responses to ICI treatment, the nonresponder group (NR, *n* = 17) exhibited substantially higher levels of serum RNase1 (Figure 3E). Notably, in the responder group, the two patients who achieved complete responses following ICI treatment exhibited the lowest serum RNase1 concentrations (215.59 and 259.77 ng mL^−1^; green dots in Figure 3E). These clinical correlation data underscore the importance of further investigating the role of RNase1 in T‐cell dysfunction.

### 2.4 RNase1 Induces CD8^+^ T‐Cell Dysfunction and Reduces CD8^+^ T‐Cell Cytotoxicity against Cancer Cells In Vivo

**Figure 4:**
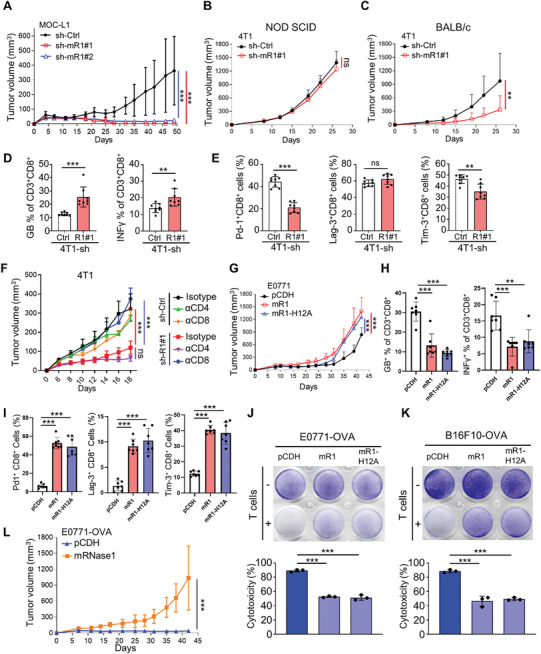
RNase1 impairs T cell cytotoxicity against cancer cells and promotes T‐cell dysfunction. A) Mouse RNase1‐knockdown (sh‐mR1#1 and #2) and control (Ctrl) MOC‐L1 cells (5 × 10^6^) were subcutaneously inoculated into C57BL/6 mice (*n* = 8 mice per group). The tumor volume was measured. B) 4T1‐sh‐Ctrl or sh‐R1#1 cells (5 × 10^4^) were orthotopically injected into NOD SCID mice (*n* = 10 mice per group). The tumor volume was measured. C) Mouse RNase1‐knockdown (sh‐mR1#1) and control (sh‐Ctrl) 4T1 cells (5 × 10^4^) were orthotopically injected into BALB/c mice (*n* = 8 mice per group). The tumor volume was measured. D) The percentage of CD3^+^CD8^+^ T cells‐expressing granzyme B (GB) or IFNγ in 4T1‐sh‐Ctrl and sh‐R1#1 tumor tissues from mice according to flow cytometry analysis (*n* = 8 independent tissue samples). E) The percentage of CD3^+^CD8^+^ T cells‐expressing Pd‐1, Lag‐3, or Tim‐3 in 4T1‐sh‐Ctrl and sh‐R1#1 tumor tissues from mice according to flow cytometry analysis (*n* = 8 independent tissue samples). F) sh‐mR1#1 and sh‐Ctrl 4T1 cells (1 × 10^5^) were orthotopically injected into BALB/c mice treated with isotype, anti‐CD4, or anti‐CD8 antibodies every 3 d (*n* = 6 mice per group). The tumor volume was measured. G) Mouse RNase1‐expressing (mR1), enzyme‐dead mouse RNase1‐expressing (mR1‐H12A), and control (pCDH) E0771 cells (2 × 10^5^) were orthotopically injected into C57BL/6 mice (*n* = 10 mice per group). The tumor volume is measured. H) The percentage of CD3^+^CD8^+^ T cells‐expressing GB or IFNγ in E0771‐Ctrl, mR1, and mR1‐H12A tumor tissues from mice according to flow cytometry analysis (*n* = 7 independent tissue samples). I) The percentage of CD3^+^CD8^+^ T cells‐expressing Pd‐1, Lag‐3, or Tim‐3 in E0771‐Ctrl, mR1, and mR1‐H12A tumor tissues from mice according to flow cytometry analysis (*n* = 7 independent tissue samples). J) The analysis of T‐cell cytotoxicity against E0771‐pCDH, mR1, or mR1‐H12A cells expressing MHC class I specific epitope of OVA (E0771‐OVA) by OT‐1 T cells. Three independent experiments with three technical replicates were carried out. K) The analysis of T‐cell cytotoxicity against B16F10‐pCDH, mR1, or mR1‐H12A cells expressing MHC class I specific epitope of OVA (B16F10‐OVA) by OT‐1 T cells. Three independent experiments with three technical replicates were carried out. L) OT‐1 TCR transgenic mice were orthotopically injected with 1 × 10^6^ cells of E0771‐pCDH or mR1 cells expressing MHC class I specific epitope of OVA, *n* = 6 for pCDH group and *n* = 5 for mR1 group. Data are presented as mean ± SD, **p, 0.001–0.01, ***p < 0.001, and ns, not significant by D,E,H–K) two‐sided unpaired Student's *t*‐test or A–C,F,G,L) an ANOVA test.

We next examined the impact of RNase1 on cancer growth and T‐cell activity in vivo by using syngeneic mouse models of HNSC and breast cancer. In one HNSC (MOC‐L1, a mouse oral carcinoma cell line) and three breast cancer (4T1, E0771, and EMT6) mouse cell lines, MOC‐L1 and 4T1 cells expressed higher mRNA levels of RNase1 than E0771 and EMT6 cells (Figure S3A, Supporting Information). Therefore, we generated knockdown control (sh‐Ctrl) and mouse RNase1 knockdown (sh‐mR1#1 and sh‐mR1#2) MOC‐L1 cells for the syngeneic mouse model of HNSC (Figure S3B, Supporting Information). Notably, the MOC‐L1‐sh‐mR1#1 and MOC‐L1‐sh‐mR1#2 cells exhibited remarkable cancer regression compared with MOC‐L1‐sh‐Ctrl cells (**Figure** **4**A). To obtain mouse cancer tissues for further analysis of T‐cell activity in TME and distinguish whether RNase1‐promoting cancer growth is from its immunosuppressive function, we knocked down RNase1 in 4T1 cells (Figure S3C, Supporting Information) and compared the cancer growth between 4T1‐sh‐Ctrl and 4T1‐sh‐mR1#1 cells in both immunocompetent and non‐obese diabetic severe combined immune deficiency (NOD SCID) mice. Notably, knockdown of mouse RNase1 resulted in reduced cancer growth only in immunocompetent but not in NOD SCID mice (Figure 4B,C), suggesting that RNase1‐induced T‐cell dysfunction may contribute to cancer progression. Subsequent analysis of mouse cancer tissues through flow cytometry revealed that RNase1 knockdown in 4T1 cells increased the proportion of CD8^+^ T cells expressing effector cytokines, including granzyme B (GB) and IFN‐γ (Figure 4D), and reduced expression of ICs such as Pd‐1 and Tim‐3 (Figure 4E) on CD8^+^ T cells in the TME. However, we did not observe these effects in CD4^+^ T cells except for the reduction of Pd‐1 in cancer tissues from 4T1‐sh‐mR1#1 cells compared to that from 4T1‐sh‐Ctrl cells (Figure S3D,E, Supporting Information). To further confirm the effect of RNase1 on T cell subsets in vivo, CD4^+^ or CD8^+^ T cells were neutralized with antibodies in mice bearing 4T1‐sh‐Ctrl or 4T1‐sh‐mR1#1 tumors. Depletion of CD8^+^ T cells (blue) abrogated tumor regression in 4T1‐sh‐mR1#1 tumor compared to the isotype‐treated group (red). However, depletion of CD4^+^ T cells (purple) in 4T1‐sh‐mR1#1 tumor did not significantly alter tumor growth (Figure 4F). These data supported that RNase1 may increase dysfunctional CD8^+^ T cells (but not CD4^+^ T cells) in vivo and result in increased tumorigenicity.

Subsequently, we established E0771 breast cancer cells expressing mouse RNase1 (mR1) and a variant lacking ribonucleolytic activity (mR1‐H12A; Figure S3F, Supporting Information) and inoculated them into either immunocompetent or immune‐deficient NOD SCID mice. We verified the absence of ribonucleolytic activity in mR1‐H12A compared with in mR1 (Figure S3G, Supporting Information). Consistently, the data from the immunocompetent mice but not the NOD SCID mice revealed that expression of either mR1 or R1‐H12A in E0771 cells promoted cancer growth (Figure 4G and Figure S3H, Supporting Information). Furthermore, expression of mR1 or mR1‐H12A in E0771 cells not only reduced the levels of GB and IFN‐γ secreted by CD8^+^ T cells but also upregulated the levels of Pd‐1, Lag‐3, and Tim‐3 on CD8^+^ T cells in the TME (Figure 4H,I). These findings further support the notion that RNase1 mediates T‐cell dysfunction independently of its enzymatic activity, contributing to an immunosuppressive TME. To further elucidate T‐cell dysregulation by RNase1, we performed MHC class I‐restricted, ovalbumin‐specific, CD8^+^ (OT‐I) T cell killing assays and used the OT‐I T cell receptor (TCR) transgenic mouse model. We engineered E0771 breast cancer cells (E0771‐OVA (ovalbumin)) and B16F10 melanoma cells (B16F10‐OVA) to express the major histocompatibility complex (MHC) class I specific epitope of OVA (Figure S3I,J, Supporting Information). Stable cell lines expressing either mR1 or mR1‐H12A were generated for use in OT‐I T‐cell killing assays (Figure S3K,L, Supporting Information). Expression of either mR1 or mR1‐H12A in both E0771‐OVA and B16F10‐OVA cells reduced OT‐I T‐cell cytotoxicity against cancer cells (Figure 4J,K). Moreover, E0771‐OVA‐control (pCDH) or mR1 stable cells were injected into OT‐I TCR transgenic mice. Compared to control cells, mouse RNase1 expression in E0771‐OVA cells led to a dramatically increased cancer growth (Figure 4L). These findings collectively suggest that secretory RNase1 induces CD8^+^ T‐cell dysfunction by impairing effector cytokine production, enhancing IC expression, and reducing T‐cell cytotoxicity, thereby contributing to an immunosuppressive TME.

### 2.5 Internalization of RNase1 into T Cells Is Critical for T‐Cell Regulation

**Figure 5:**
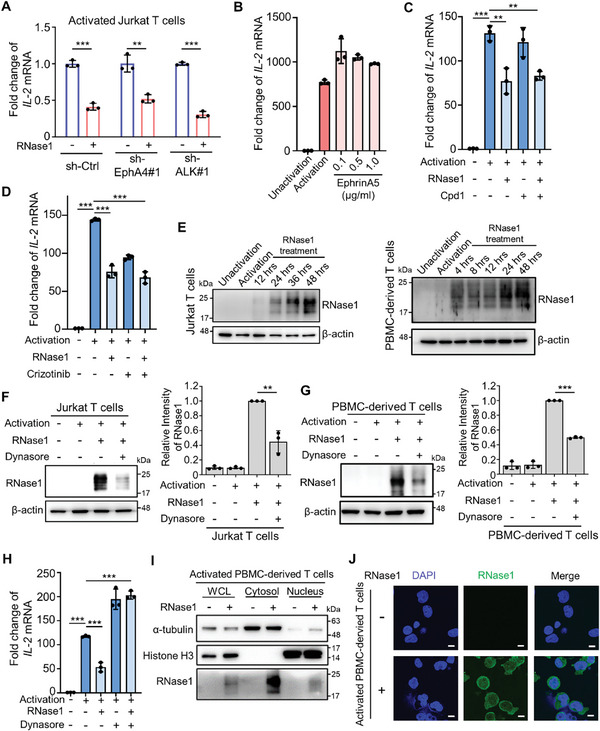
RNase1‐induced T‐cell dysregulation may require the internalization of RNase1 into T cells. A) Quantitative RT‐PCR analysis of *IL‐2* mRNA expression in EphA4‐knockdown (sh‐EphA4#1), ALK‐knockdown (sh‐ALK#1), or control (sh‐Ctrl) activated Jurkat T cells treated without or with RNase1 (1 µg mL^−1^) for 48 h. Representative data from three independent experiments (each experiment contains three technical replicates). B) Quantitative RT‐PCR analysis of *IL‐2* mRNA expression in unactivated Jurkat T cells and activated Jurkat T cells treated without or with different concentrations of recombinant EphrinA5 as indicated for 48 h. Representative data from three independent experiments (each experiment contains three technical replicates). C) Quantitative RT‐PCR analysis of *IL‐2* mRNA expression in unactivated Jurkat T cells and activated Jurkat T cells treated without or with RNase1 (1 µg mL^−1^), Cpd1 (10 µm), or RNase1 combined to Cpd1 for 48 h. Representative data from three independent experiments (each experiment contains three technical replicates). D) Quantitative RT‐PCR analysis of *IL‐2* mRNA expression in unactivated Jurkat T cells and activated Jurkat T cells treated without or with RNase1 (1 µg mL^−1^), crizotinib (0.5 µm), or RNase1 combined to crizotinib for 48 h. Representative data from three independent experiments (each experiment contains three technical replicates). E) Left: Western blot analysis of RNase1 in unactivated Jurkat T cells and activated Jurkat T cells treated without or with RNase1 (1 µg mL^−1^) for different time‐points as indicated. Right: Western blot analysis of RNase1 in unactivated PBMC‐derived T cells and activated PBMC‐derived T cells treated without or with RNase1 (1 µg mL^−1^) for different time‐points as indicated. β‐actin served as a loading control. Representative data from three independent experiments. F) Left: Western blot analysis of RNase1 in unactivated Jurkat T cells and activated Jurkat T cells treated without or with RNase1 (1 µg mL^−1^) or RNase1 combined with dynasore (10 µm for 1 h treatment) for 48 h. Right: Quantitative results of Western blot of RNase1 in the left panel of (F). Quantitative data were analyzed from three independent experiments. G) Left: Western blot analysis of RNase1 in unactivated PBMC‐derived T cells and activated PBMC‐derived T cells treated without or with RNase1 (1 µg mL^−1^) or RNase1 combined with dynasore (10 µm for 1 h treatment) for 48 h. β‐actin served as a loading control. Right: Quantitative results of Western blot of RNase1 in the left panel of (G). Quantitative data were analyzed from three independent experiments. H) Quantitative RT‐PCR analysis of *IL‐2* mRNA expression in unactivated Jurkat T cells and activated Jurkat T cells treated without or with RNase1 (1 µg mL^−1^), dynasore (10 µm for 1 h treatment), or RNase1 combined with dynasore (20 µm for 1 h treatment) for 48 h. Representative data from three independent experiments (each experiment contains three technical replicates). I) Western blot analysis of the whole cell lysate (WCL), cytoplasmic, and nuclear fractions in activated Jurkat T cells (JA) and 1 µg mL^−1^ RNase1‐treated activated Jurkat T cells (JAR). Representative data from three independent experiments. J) Immunocytochemistry staining of RNase1 in activated PBMC‐derived T cells treated without or with RNase1 (1 µg mL^−1^) for 48 h. Nuclei were counterstained with DAPI. Representative images of *n* = 2 independent replicates. Scale bar: 10 µm. Data represent mean ± S.D. **p, 0.001–0.01, and ***p < 0.001 by two‐sided unpaired Student's *t*‐test.

Considering that RNase1‐induced T‐cell dysfunction is independent of its enzymatic activity and that RNase1 functioning as a ligand of tyrosine kinase receptors, ephrin A4 (EphA4) and anaplastic lymphoma kinase (ALK), to activate their downstream signaling is also enzymatic activity independent,^[^ ^6^, ^11^ ^]^ we sought to determine whether RNase1‐activated EphA4 and ALK signaling might contribute to T‐cell dysregulation. We knocked down EphA4 or ALK in Jurkat T cells (Figure S4A,B, Supporting Information) and analyzed the mRNA levels of IL‐2 in activated Jurkat‐sh‐Ctrl, Jurkat‐sh‐EphA4#1, and Jurkat‐shALK#1 cells treated with or without RNase1. RNase1 treatment still led to a significant reduction in IL‐2 mRNA expression in activated Jurkat‐sh‐EphA4#1 and Jurkat‐shALK#1 cells compared with that in the untreated control (**Figure** **5**A). In addition, our experiments revealed that activation of EphA4 by its traditional ligand EphrinA5 did not lead to a decrease in IL‐2 mRNA in activated Jurkat T cells (Figure 5B). Furthermore, treatment with an EphA4 inhibitor (Cpd1) or an ALK inhibitor (crizotinib) did not alter the RNase1‐mediated repression of IL‐2 mRNA levels in activated Jurkat T cells (Figure 5C,D). In addition, we did not find a clear colocalization of RNase1 with EphA4 or ALK in activated T cells treated with RNase1 by immunocytochemistry (ICC) staining (Figure S4C, Supporting Information). These data did not support that RNase1‐induced T‐cell dysfunction was mediated through ligand/receptor interactions with EphA4 or ALK. Studies have reported that some RNases can internalize and translocate into cell cytoplasm through endocytosis.^[^ ^12^, ^20^ ^]^ Encouraged by these findings, we asked whether RNase1 might internalize into T cells and interact with specific proteins involved in T‐cell activity. In line with this notion, we observed the presence of RNase1 inside the activated Jurkat and PBMC‐derived T cells following treatment with recombinant RNase1 (Figure 5E). Furthermore, inhibition of endocytosis by using the inhibitor dynasore significantly reduced RNase1 internalization into activated Jurkat and PBMC‐derived T cells (Figure 5F,G). The inhibition of RNase1 internalization by dynasore neutralized the decrease in IL‐2 mRNA levels induced by RNase1 treatment (Figure 5H). We also detected that the majority of RNase1 was localized in the cytoplasm of activated T cells from the sodium dodecyl sulfate polyacrylamide gel electrophoresis (SDS‐PAGE) in which equal amounts of sample proteins were loaded after a cell fractionation assay (Figure 5I). The results of ICC staining also showed that RNase1 was concentrated in the cytoplasm of activated T cells (Figure 5J). Collectively, these results suggest that the entry of RNase1 into T cells is a crucial step in dysregulation of T‐cell activity and the majority of RNase1 stays in the cytoplasm.

### 2.6 RNase1 Interacts with STAT1 to Inhibit T Cell‐Mediated Cancer Cell Killing

**Figure 6:**
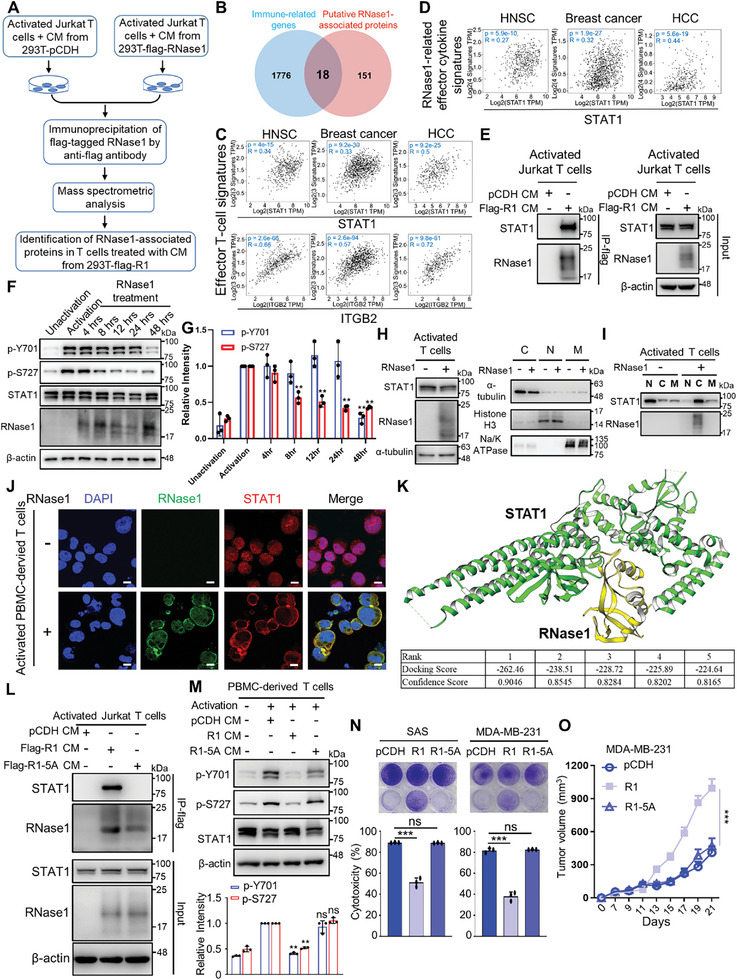
RNase1 hijacks STAT1 to inhibit T‐cell cytotoxicity. A) A schematic outline for the identification of putative RNase1‐associated proteins by IP‐MS analysis using activated‐Jurkat T cells treated with CM from 293T cell‐expressing flag‐tagged RNase1 (flag‐R1, *n* = 2) compared to that from vector control (pCDH). B) The Venn diagram for the intersection of immune‐related genes (blue color) in the ImmPort database and putative RNase1 associated proteins from the result of IP‐MS analysis (red color). C) Correlation analysis between STAT1 versus effector T cell signatures (upper panel) and ITGB2 versus effector T cell signatures (lower panel) in patient tissues of HNSC, breast cancer, and HCC from the GEPIA2 database. D) Correlation analysis between STAT1 and RNase1‐related effector cytokine signatures in patient tissues of HNSC, breast cancer, and HCC from the GEPIA2 database. E) Activated Jurkat T cells treated with CM from 293T‐flag‐R1 or pCDH cells were subjected to immunoprecipitation followed up Western blot analysis with flag antibody‐conjugated magnetic beads. β‐actin was a loading control. Representative results from three independent experiments. F) Western blot analysis of p‐Y701‐STAT1, p‐S727‐STAT1, STAT1, and RNase1 in unactivated PBMC‐derived T cells and activated PBMC‐derived T cells treated without or with RNase1 (1 µg mL^−1^) for different time‐points as indicated. β‐actin served as a loading control. Representative results from three independent experiments. G) Quantitative results of Western blot of p‐Y701‐STAT1 and p‐S727‐STAT1 in (C). Quantitative data were analyzed from three independent experiments. H) Left: Western blot analysis of STAT1 and RNase1 in activated T cells treated without or with RNase1 (1 µg mL^−1^) for 48 h. α‐tubulin served as a loading. Right: Western blot analysis of the nuclear (N), cytoplasmic (C), and membrane (M) fractions in activated T cells with or without 1 µg mL^−1^ RNase1 treatment for 48 h. Representative results from three independent experiments. I) Western blot analysis of STAT1 and RNase1 in the nuclear, cytoplasmic, and membrane fractions from activated T cells with or without 1 µg mL^−1^ RNase1 treatment for 48 h. Representative results from three independent experiments. J) Immunocytochemistry staining of RNase1 and STAT1 in activated PBMC‐derived T cells treated without or with RNase1 (1 µg mL^−1^) for 48 h. Nuclei were counterstained with DAPI. Representative images of *n* = 2 independent replicates. Scale bar: 10 µm. K) Upper: The RNase1/STAT1 binding model with highest scores. This model was proposed by the HDOCK server. The docking and confidence scores of this model are 262.45 and 0.90, indicating that the predicted binding is reasonable. Lower: The docking and confidence scores of the top 5 RNase1/STAT1 models. L) Activated Jurkat T cells treated with CM from 293T‐flag‐R1, flag‐R1‐5A (the amino‐acid residues of 87‐TNGSR‐91 on RNase1 substituted to five alanine), or pCDH cells were subjected to immunoprecipitation followed up Western blot analysis with flag antibody‐conjugated magnetic beads. β‐actin was a loading control. Representative results from three independent experiments. M) Upper: Western blot analysis of p‐Y701‐STAT1, p‐S727‐STAT1, and STAT1 in unactivated PBMC‐derived T cells and activated PBMC‐derived T cells treated with CM from 293T‐flag‐R1, flag‐R1‐5A, or pCDH cells. β‐actin served as a loading control. Representative results from three independent experiments. Lower: Quantitative results of Western blot of p‐Y701‐STAT1 and p‐S727‐STAT1 in the upper panel of (M). Quantitative data were analyzed from three independent experiments. N) Representative images and quantitative results of T cell‐mediated cancer cell killing assay. Left: SAS‐pCDH, R1, and R1‐5A cells (5 × 10^4^ cells) cocultured with or without activated PBMC‐derived T cells for 48 h were subjected to crystal violet staining to determine T‐cell cytotoxicity. SAS to T‐cell ratio, 1:6. Right: MDA‐MB‐231‐pCDH, R1, and R1‐5A cells (2 × 10^4^ cells) cocultured with or without activated PBMC‐derived T cells for 48 h were subjected to crystal violet staining to evaluate T‐cell cytotoxicity. MDA‐MB‐231 to T‐cell ratio, 1:5. Three independent experiments with three technical replicates were carried out. O) MDA‐MB‐231‐pCDH, R1, and R1‐5A cells (5 × 10^6^) were orthotopically injected into NPG mice received with 1 × 10^7^ CD8^+^ T cells isolated from activated PBMC‐derived T cells on Day 7 after tumor inoculation (*n* = 10 mice per group). The tumor volume was measured. Data represent mean ± S.D. **p, 0.001–0.01, ***p < 0.001, and ns, not significant by G,M,N) two‐sided unpaired Student's *t*‐test or O) an ANOVA test.

**Figure 7:**
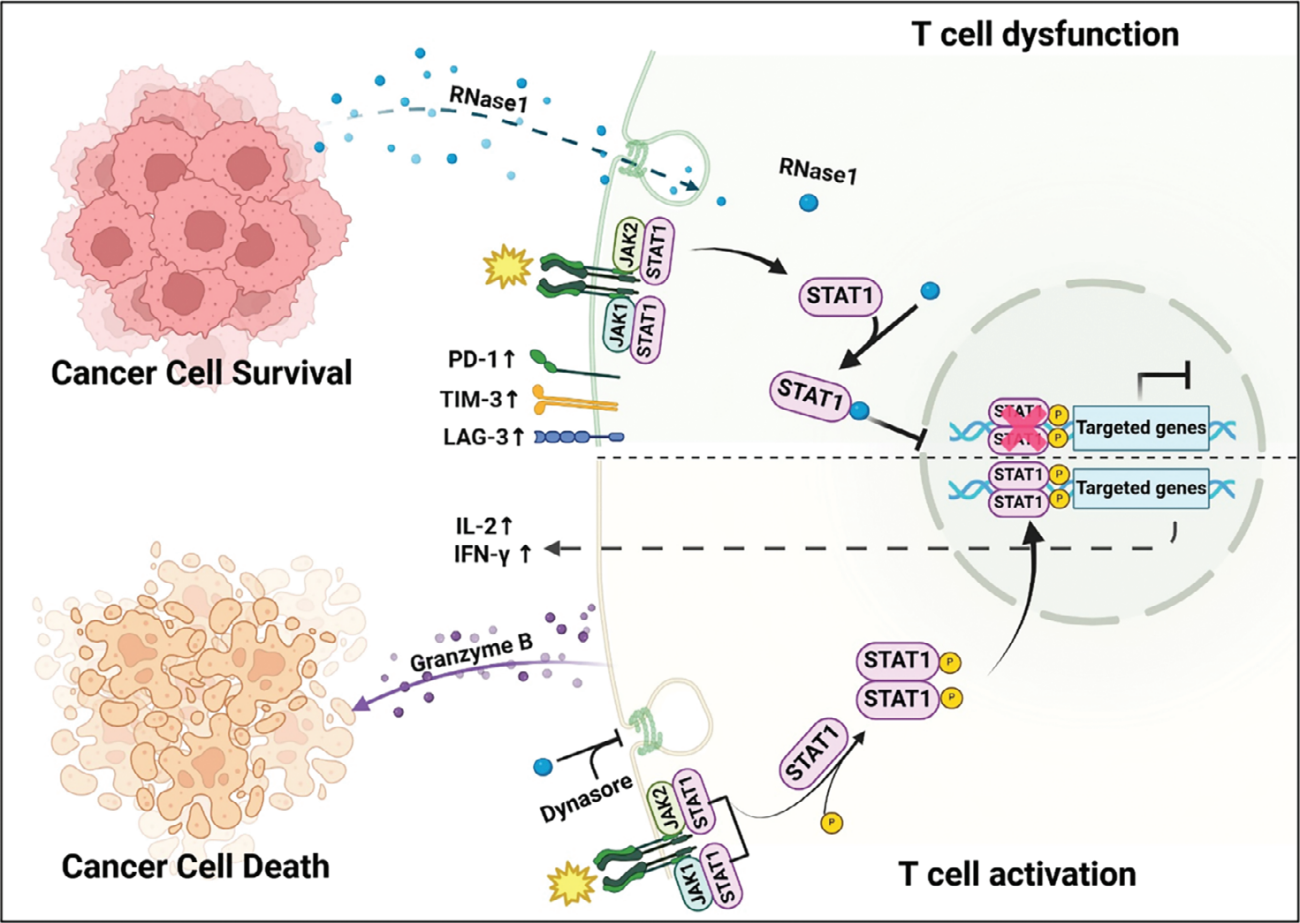
A proposed model of RNase1 as a cancer‐secreted immune checkpoint‐like protein to induce T‐cell dysfunction in the tumor microenvironment. STAT1 has been reported to promote T‐cell anticancer immunity and repress T‐cell exhaustion (bottom panel). We propose that high RNase1‐expressing cancer cells secret RNase1 to internalize into T cells through endocytosis manner. RNase1 interacts with STAT1 to repress STAT1 activation and restrict STAT1 localization in the cytoplasm. Moreover, RNase1 also increases the expression of PD‐1, TIM‐3, and LAG‐3, decreases the levels of Granzyme B, INF‐r, and IL‐2, and inhibits CD8^+^ T‐cell cytotoxicity to result in T cell dysfunction and cancer sell survival (upper panel). The figure was created in BioRender.

Next, we asked whether RNase1 may interact with T‐cell activity‐related proteins in activated T cells. To this end, we performed the immunoprecipitation (IP) of flag‐tagged RNase1, followed by MS analysis in activated‐Jurkat T cells treated with CM from 293T cells‐expressing flag‐tagged RNase1 (flag‐R1) or vector control (pCDH). Through our analysis, we identified 169 putative RNase1‐associated proteins detected exclusively in Jurkat T cells treated with CM from 293T‐flag‐R1 but not in those treated with CM from 293T‐pCDH cells (**Figure** **6**A). We then compared these 169 putative RNase1‐associated proteins with immune‐related genes (IRGs, *n*  =  1793) from the ImmPort database.^[^ ^21^ ^]^ We identified 18 out of 169 putative RNase1‐associated proteins that overlapped with IRGs (Figure 6B). Since high‐RNase1 expression enriches T‐cell exhaustion signatures in patients with HNSC, breast cancer, and HCC (Figure 3D), we further performed correlation analysis between the gene expression of these 18 proteins and effector T‐cell signatures in HNSC, breast cancer, and HCC patient samples by using the GEPIA2 database.^[^ ^22^ ^]^ STAT1 and ITGB2 out of the 18 candidates were identified to show a significant positive correlation with effector T‐cell signatures in these three cancer types (Figure 6C). ITGB2, also known as lymphocyte function‐associated antigen 1 or CD18, is integrin beta 2 which is expressed on T cell surface and involves in T cell trafficking and effector functions through the engagement with other immune cells such as antigen presenting cells.^[^ ^23^ ^]^ Since RNase1 can directly induce T‐cell dysfunction without the presence of antigen presenting cells (Figure 1), and STAT1 signaling has been known to be required for T cell activation,^[^ ^24^ ^]^ also implicating in suppressing T‐cell exhaustion to enhance T‐cell‐mediated anticancer immunity,^[^ ^25^ ^]^ we therefore chose RNase1 and STAT1 interaction to pursue first. To this end, we found that STAT1 in HNSC, breast cancer, and HCC patient tissues displayed a positive correlation with RNase1‐related effector cytokine signatures according to the analysis of TCGA dataset by using the GEPIA2 database (Figure 6D), including IL‐2, INF‐γ, IL‐6, and GM‐CSF which have been shown to be repressed by RNase1 in activated T cells (Figure 1 and Figure S1, Supporting Information). These findings suggest that RNase1 may interact with STAT1, potentially leading to the dysregulation of effector T‐cell function. To test this possibility, we first showed that RNase1 indeed interacted with STAT1 by Co‐IP assays in the RNase1 stimulated lysates (Figure 6E). Next, we examined whether RNase1 treatment represses STAT1 activation in T cells. Because STAT1 activation relies on phosphorylation at two critical sites, tyrosine 701 (Y701) and serine 727 (S727),^[^ ^26^ ^]^ we analyzed the changes in these two phosphorylation sites after RNase1 treatment in activated T cells. The results revealed a significant reduction in Y701 phosphorylation (p‐Y701) at 48 h posttreatment and a decrease in S727 phosphorylation (p‐S727) at 8 h posttreatment (Figure 6F,G). These findings indicated that RNase1 inhibited STAT1 activation in T cells and suggested that RNase1 might inhibit STAT1 activation through their interaction. Research indicates that p‐Y701 facilitates translocation of STAT1 from cytoplasm to the nucleus, where it exerts its transcriptional activity.^[^ ^27^ ^]^ Notably, in the current study, RNase1 was discovered to reduce the level of p‐Y701 STAT1 (Figure 6F) and was observed to localize in the cytosol following internalization into T cells (Figure 5I). This led us to speculate that RNase1 may sequester STAT1 in cytoplasm and thereby inhibit its function. To investigate this possibility, we conducted nucleus/cytoplasm/membrane fractionation assays to isolate nuclear, cytoplasmic, and membrane fractions from activated T cells treated with or without RNase1 (Figure 6H). Upon treatment with RNase1, the localization of STAT1 shifted from the nucleus to cytoplasm in activated T cells, indicating that RNase1 could confine STAT1 within the cytoplasm of T cells. (Figure 6I). We also observed by ICC staining that STAT1 was clearly colocalized with RNase1 and the nuclear translocation of STAT1 was blocked in activated T cells after RNase1 treatment (Figure 6J). The results of coefficient calculations, with a Pearson's correlation coefficient of 0.477, a Manders' overlap coefficient tM1 (RNase1 over STAT1) of 0.745, and a Manders' overlap coefficient tM2 (STAT1 over RNase1) of 0.737, further supported the colocalization observation (Figure S5A, Supporting Information). To validate whether disruption of the RNase1‐STAT1 interaction could restore STAT1 activation in T cells, we attempted to identify the key residues responsible for the binding between RNase1 and STAT1 through analysis of a proposed binding model. The analysis of atomic structures of RNase1 (PDBID: 1Z7X) and STAT1 (PDBID: 1YVL) in HDOCK server (a web server for protein‐protein and protein‐DNA/RNA docking based on a hybrid strategy) indicated that STAT1 received a good binding model with RNase1 (Figure 6K).^[^ ^28^ ^]^ On the basis of this model, the residues involved in the interface were proposed (Figure S5B, Supporting Information). Among these residues, a loop region in RNase1 (i.e., 87‐TNGSR‐91) was identified as potentially playing a vital role in the binding to STAT1. This RNase1 loop was located at the center of the binding interface and was in close proximity to another loop on STAT1 (i.e., 246‐ACIGGPPNAC‐255 and Figure S5C, Supporting Information). Subsequently, all five amino acids located in the RNase1 loop were mutated to alanine, and the mutated RNase1 variant (R1‐5A) was expressed in 293T (Figure S5D, Supporting Information). Compared with that in wild‐type RNase1, the R1‐5A mutant retained its enzymatic activity (Figure S5E, Supporting Information), indicating that this mutation did not disrupt the functional structure of RNase1. Interestingly, this mutated RNase1 lost its ability to bind to STAT1 (Figure 6L). Notably, we observed that this mutated RNase1 also failed to suppress the levels of p‐Y701 and S727‐STAT1 in activated T cells (Figure 6M). To assess the impact of mutated RNase1 on T‐cell cytotoxicity, stable cell lines expressing wild‐type RNase1 and the R1–5A mutant were established in SAS and MDA‐MB‐231 cells (Figure S5F,G, Supporting Information), and these cell lines were subjected to T‐cell killing assays. The results of these assays confirmed that the mutated RNase1 lost its ability to inhibit T‐cell cytotoxicity against cancer cells (Figure 6N), supporting that disrupting the RNase1‐STAT1 interaction may prevent RNase1‐induced T‐cell dysfunction. Next, we adoptively transferred purified CD8^+^ T cells into immunodeficiency Prkdc^scid^Il2rg^null^ (NPG) mice inoculated with MDA‐MB‐231‐pCDH, MDA‐MB‐231‐R1, or MDA‐MB‐231‐R1‐5A cells to evaluate the effect of disrupting the RNase1‐STAT1 interaction in CD8^+^ T cells. The results showed that R1‐5A expressed in MDA‐MB‐231 abolished the RNase1‐promoted increases in tumor volume and weight (Figure 6O and Figure S5H,I, Supporting Information), suggesting that the RNase1‐STAT1 interaction in CD8^+^ T cells may be crucial for RNase1 to inhibit CD8^+^ T‐cell cytotoxicity against cancer cells in vivo. Taken together, our findings indicate that RNase1 internalizes into T cells, interacts with STAT1, and prevents STAT1 from nuclear localization, thereby losing its ability to activate T cells (**Figure** **7**).

## 3 Discussion

Consistent with the findings of the current study, research has indicated that administration of RNase1 in mice leads to a reduction in the secretion of proinflammatory cytokines.^[^ ^29^ ^]^ Moreover, we previously revealed RNase1 to be involved in conferring resistance to Nivolumab, an anti‐PD‐1 antibody, by promoting the polarization of macrophages toward a tumor‐associated phenotype in HCC.^[^ ^11e^ ^]^ These observations suggest that RNase1 might play an immunosuppressive role in the TME. In the current study, our unexpected findings revealed that direct treatment of T cells with recombinant RNase1 resulted in decreased expression levels of IL‐2, INF‐γ, IL‐6, and GM‐CSF (Figure 1E–G and Figure S1, Supporting Information). Furthermore, we demonstrated that RNase1 increased the expression of immune inhibitory checkpoint proteins (Figure 1H,I) and compromised CD8^+^ T‐cell cytotoxicity in vitro (Figure 2H–J), and in vivo experiments demonstrated that RNase1 inhibited CD8^+^ T‐cell activity, thereby promoting breast cancer growth (Figure 4G–I). These findings underscore the potential contribution of the secretory RNase1 to T‐cell dysfunction and exhaustion within an immunosuppressive TME.

T‐cell dysfunction is associated to an exhausted state which is characterized by high levels of multiple ICs and impaired effector functions, including T‐cell cytotoxicity and the production of effector cytokines.^[^ ^30^ ^]^ Here, the in vitro and in vivo data consistently implied that RNase1 led to a decrease of effector cytokines (IL‐2 and INF‐γ in vitro; GB and INF‐γ in vivo) and an increase of multiple ICs (PD‐1, LAG‐3, and TIM‐3) in CD8^+^ T cells, which may contribute to CD8^+^ T‐cell dysfunction during cancer progression (Figure 2B,G and Figure 4D–I). Because IL‐2 and INF‐γ production during T‐cell activation was repressed in the presence of RNase1 (Figure 1E–G), we speculate that RNase1 would also interfere with T‐cell activation, thereby inhibiting T cell activity. However, RNase1 treatment did not affect cell proliferation of CD4^+^ and CD8^+^ T cells (Figure S2B, Supporting Information). Therefore, RNase1 should promote a state of T‐cell exhaustion and impair T‐cell activation and effector function to inhibit CD8^+^ T‐cell cytotoxicity, thereby promoting tumor growth, which we observed in this study (Figure 2J and Figure 6O). Interestingly, increased multiple ICs and decreased effector cytokines in CD4^+^ T cells were induced by RNase1 in vitro (Figure 2A,F), but not in vivo (Figure S3D,E, Supporting Information). We speculate that the effects of RNase1 in CD4^+^ T cells may be short‐term and undetectable in long‐term mouse models. Nonetheless, the function and differentiation of CD4^+^ T‐cells might also play a role in T‐cell cytotoxicity. For example, a deficiency in CD4^+^ T cell help leads to T‐cell dysfunction.^[^ ^31^ ^]^ Therefore, the short‐term effects of RNase1 on CD4^+^ T‐cell function and differentiation warrant further investigation.

The present study also suggested a novel regulatory mechanism governing STAT1 activation in T cells. Although several studies have underscored the crucial role of STAT1 in T‐cell function^[^ ^24a^ ^]^ and its ability to repress T‐cell exhaustion and thereby promote T‐cell‐mediated anticancer immunity,^[^ ^25^ ^]^ the regulatory mechanism is not fully elucidated. Our findings revealed that secreted RNase1 infiltrates T cells and engages with STAT1, resulting in diminished levels of p‐Y701 and p‐S727 STAT1 and consequently impairing CD8^+^ T‐cell cytotoxicity (Figure 6F,N,O). These results underscore the pivotal role of STAT1 in maintaining T‐cell function and suggest that disrupting the RNase1‐STAT1 interaction could mitigate RNase1‐induced T‐cell dysfunction in the TME. Notably, the time points of inhibition by RNase1 differ between p‐Y701 and p‐S727 STAT1; the reduction in p‐S727 STAT1 precedes that in p‐Y701 STAT1 (Figure 6G). This intriguing observation could be attributed to the sequential nature of STAT1 phosphorylation during T‐cell activation. Janus kinases (JAKs) phosphorylate STAT1 at Y701, which enables STAT1 to form a homodimer/heterodimer, translocate to the nucleus, and bind to DNA.^[^ ^32^ ^]^ S727 phosphorylation of STAT1 is essential for enabling the full transcriptional activity and biological function of STAT1. Y701 phosphorylation and nuclear translocation are both necessary for STAT1 S727 phosphorylation induced by INF.^[^ ^26^ ^]^ In addition, cyclin‐dependent kinase 8 (CDK8) was demonstrated to phosphorylate STAT1 at S727.^[^ ^33^ ^]^ Combined with our current data, these observations imply that RNase1 may prevent the recruitment of kinases such as JAKs or CDK8 to STAT1 and restrict STAT1 within the cytoplasm of T cells. Future studies should elucidate the structural basis of the RNase1‐STAT1 interaction and explore the mechanism by which RNase1 binding induces STAT1 inactivation to enable development of relevant clinical interventions.

In our study published in the early of 2024 by Liu et al., RNase1 has been found to cause an immunosuppressive TME by promoting M2‐like tumor‐associated macrophage (TAM) polarization through its interaction with ALK and activation of the ALK/STAT3 pathway in HCC,^[^ ^11e^ ^]^ supporting RNase1 functions as an immunosuppressive role in TME. In the previous study, the immune cell profile was analyzed through cytometry by time of flight analysis in HCC mouse tumor tissues and an increased M2‐like TAM in high RNase1‐expressing tumors was observed. It was concluded that these RNase1‐induced M2‐like TAMs may prevent CD8^+^ T‐cell infiltration to create an immunosuppressive TME in HCC. However, analysis of RNase1 expression and immune cell infiltration profiles from the TCGA database in this previous study showed that in addition to macrophage infiltration, T cell infiltration is also positively correlated with RNase1 levels in HCC patients.^[^ ^11e^ ^]^ We further carried out the same analysis in breast cancer and HNSC patients and discovered that the infiltration of T cells including CD8^+^ T cells positively correlates with RNase1 levels (Figure S6 and Table S1,S2, Supporting Information). Therefore, the CD8^+^ T‐cell dysfunction directly induced by RNase1 may play a vital role to repress anticancer immunity in T cell‐infiltrating tumors. Interestingly, the previous study observed that RNase1 treatment reduced the level of p‐Y701 STAT1 in macrophage to repress M1‐like macrophage polarization with an unknown mechanism.^[^ ^11e^ ^]^ Here, we found that RNase1‐STAT1 interaction decreases the level of p‐Y701 STAT1 in T cells (Figure 6F,G), which is consistent with the previous observation of STAT1 inhibition by RNase1 in macrophage and provides a rationale for exploring how RNase1 inhibits M1‐like macrophage polarization through STAT1 activity.

Of note, the study published by Wang et al. has shown that RNase1 promotes CD4^+^ T‐cell activation to repress breast cancer growth through the activation of EphA4 on T cells.^[^ ^34^ ^]^ In this previous study, ectopic overexpression of RNase1 in 4T1 cells inoculated into mice reduced tumor growth by an increased population of CD69‐positive CD4^+^ T cells. However, the current study revealed that knockdown of RNase1 in 4T1 cells decreased tumor growth (Figure 4C) and did not affect CD4^+^ T‐cell activity in vivo (Figure S3D,E, Supporting Information). The discrepancy between the two experiments may be attributed to inappropriate high expression of RNase1 in 4T1 cells due to their already elevated endogenous levels of RNase1 (Figure S3A, Supporting Information). It has been demonstrated that increased RNase1 expression in serum or plasma of cancer patients is mainly sourced from tumor cells.^[^ ^6^, ^11^ ^]^ To test whether RNase1‐overexpressing 4T1 tumor possesses a supraphysiological level of RNase1 in serum of mice, BALB/c mice were implanted with 4T1‐pCDH, R1, sh‐Ctrl, and sh‐mR1#1 cells and mouse serum were collected after tumor inoculation for 20 d. Compared with the plasma RNase1 level of 0.52 µg mL^−1^ in mice with RNase1‐overexpressing HCC tumor reported in a previous study,^[^ ^11e^ ^]^ the serum RNase1 level (1.37 µg mL^−1^) of mice in the 4T1‐R1 group indeed showed an abnormally high concentration (Figure S7, Supporting Information). In addition, the activity of Jurkat T cells was repressed by RNase1 due to the decrease of an effector cytokine IL‐2 and the increases of three different ICs in the current study. In contrast, the same concentration of RNase1 treatment increased a small fraction of CD69^+^ Jurkat T cells, which were considered as activated T cells based on the fact that CD69 is a traditional T cell activation marker^[^ ^35^ ^]^ in the study of Wang et al. However, studies have shown that CD69 is linked to T‐cell exhaustion and increased tumor progression.^[^ ^25^, ^36^ ^]^ Therefore, using CD69 as the only T cell activation marker may not be sufficient to determine whether CD4^+^ Jurkat T cells are activated. To ensure accuracy, in the current study, we used multiple markers including cytokine IL‐2 as an activation marker and the three different ICs, PD‐1, LAG‐3 and TIM‐3 as exhaustion markers which are more reliable. More interestingly, the data in Figure 5A–C indicated that RNase1‐induced T cell dysfunction is independent of EphA4 signaling. Nevertheless, the study of Wang et al. claimed that EphA4 signaling mediates RNase1‐induced CD4^+^ T‐cell activation. This conclusion could be overinterpreted from the experimental results. First, treatment of an EphA4 inhibitor Cpd1 did not neutralize the tumor regression induced by RNase1 overexpression and Cpd1 treatment only could repress 4T1 tumor growth in the Figure 1 of this previous study. These data suggest that tumor regression by RNase1 overexpression is independent to EphA4 signaling. Secondly, there is no direct evidence to support that RNase1 overexpression or treatment increases the population of CD69‐positive CD4^+^ T cells and T‐cell cytotoxicity through EphA4. The previous study only showed that EphA4 may be expressed in T cells and PBMCs by online database and flow cytometry analysis. Although the treatment of recombinant EphA4 protein could reduce T‐cell cytotoxicity against RNase1‐overexpressing breast cancer cells, these results cannot fully support RNase1‐induced CD4^+^ T‐cell activation through RNase1‐EphA4 axis due to the other functions or off‐target effects of recombinant EphA4 protein.

Despite some discrepancies between our current study and that of Wang et al. could be explained, we cannot ignore that aberrant overexpression of RNase1 may promote T‐cell cytotoxicity in vitro and in vivo. We speculate that aberrant overexpression of RNase1 may promote the differentiation of a specific CD4^+^ cytotoxic T cells with CD69 expression because RNase1 increases a small population of CD69‐positive CD4^+^ T cells and boost an immediate killing effect in vitro in the study of Wang et al. Preclinical and clinical studies have reported that certain intratumoral CD4^+^ T cells possess cytotoxic activity to directly mediate cancer cell death.^[^ ^37^ ^]^ In order to accurately develop RNase1 as a serum biomarker or therapeutic target in clinical practice to determine and boost T‐cell activity in tumors, further careful investigations are necessary to elucidate the complex role of RNase1 in the differentiation and activation of CD4^+^ T cells. One notable contribution of the current study is its finding that patients with HNSC who responded to ICI therapies exhibited lower serum levels of RNase1 compared with those of nonresponders (Figure 3E). A similar observation was reported in patients with HCC treated with Nivolumab.^[^ ^11e^ ^]^ Because the efficacy of ICIs and T‐cell‐based cancer immunotherapies relies on optimal activity of T cells in the TME^[^ ^38^ ^]^ and CD8^+^ T cells are the major subsets for anticancer immunity,^[^ ^39^ ^]^ our findings lay the groundwork for validating whether serum RNase1 can serve as a serum biomarker for predicting the efficacy of CD8^+^ T‐cell‐based cancer immunotherapies.

Although some RNases with ribonucleolytic activity exhibit direct cytotoxicity to kill cancer cells,^[^ ^40^ ^]^ wild‐type RNase1 has been shown to be noncytotoxic to cancer cells.^[^ ^41^ ^]^ Because the ribonucleolytic activity of RNases is inhibited by RNase inhibitors (RIs), which block the digestion of intracellular RNAs by RNases,^[^ ^12^, ^42^ ^]^ most anticancer RNases must be engineered to evade RI binding. In this study, 1 µg mL^−1^ wild‐type RNase1 also did not show significant cytotoxicity and intracellular RNA digestion in T cells and breast cancer cells in vitro (Figure S2A–E,K–M, Supporting Information). It is possible that endogenous RIs are sufficient to inhibit the enzymatic activity of RNase1 under both physiological and pathological conditions, or that cancer cells increase RI expression to repress intracellular RNA digestion by RNase1. Further in‐depth exploration on this topic is warranted.

Although studies have reported catalytic‐independent roles of the hRNase A superfamily as ligands for membrane receptors, the current study further revealed that RNase1 disrupts T‐cell function by internalizing and hijacking STAT1 within the cytoplasm of T cells. Notably, this mechanism of RNase1‐induced T‐cell dysfunction operates independently of the two known RNase1/receptor‐mediated pathways,^[^ ^6^, ^11^ ^]^ suggesting the existence of a novel ribonucleolytic activity‐independent mechanism for RNase1. Therefore, our findings introduce a fresh perspective, indicating that further exploring the binding partners of the hRNase A superfamily after their internalization into cells for uncovering their previously unrecognized biological functions. This includes investigating those ribonucleolytic activity‐deficient RNases, whose biological roles in ribonuclease biology remain unclear. Identifying additional RNase‐binding proteins within cells and elucidating their effects may reveal the biological significance and translational potential of secreted RNases as biomarkers or therapeutic targets in human diseases.

## 4 Experimental Section

### Study Approval

Serum of HNSC patients receiving immune checkpoint inhibitors for ELISA analyses were obtained from Taipei Veterans General Hospital. The study was approved by the Institutional Review Board of Taipei Veterans General Hospital (Institutional Review Board certificate No. 2022‐04‐013BCF). Serum samples were collected and used with the full and informed consent policy. All animal studies were conducted in accordance with the requirements and guidelines approved by the China Medical University Animal Care and Use Committee (Protocol No. CMUIACUC‐2021‐038 and CMUIACUC‐2022‐123).

### Cell Culture and Treatment

The cell lines 293T (CRL‐3216), Jurkat (TIB‐152), FaDu (HTB‐43), MDA‐MB‐231 (HTB‐26), A549 (CCL‐185), H1299 (CRL‐5803), Hela (CCL‐2), 4T1 (CRL‐2539), E0771 (CRL‐3461), EMT6 (CRL‐2755), and B16‐F10 (CRL‐6475) were obtained from American Type Culture Collection (Manassas, VA, USA). KPL4 cells were obtained from MD Anderson Cancer Center (Houston, TX, USA). SAS cells were purchased from Bioresource Collection and Research Center of Taiwan (Hsinchu City, Taiwan). OECM‐1 (a human oral squamous carcinoma cell line) and MOC‐L1 cells were kindly acquired from Dr. Kuo‐Wei Chang (National Yang Ming Chiao Tung University, Taipei, Taiwan). All cell lines were tested mycoplasma negative and validated by short‐term repeat DNA fingerprinting. Jurkat, FaDu, 4T1, OECM‐1, H1299, and PBMC‐derived T cells were maintained in Roswell Park Memorial Institute (RPMI) 1640 medium (#SH30255.02; Hyclone) supplemented with 10% fetal bovine serum (#SH30396.03; Hyclone). Other cell lines used in this study were cultured in Dulbecco's modified Eagle's medium (#SH30243.02; Hyclone) supplemented with 10% fetal bovine serum. Recombinant RNase1 (#13468‐H08H‐100, Sino Biological Inc.) treatment was carried out at a concentration of 1 µg mL^−1^ or indicated concentrations for the indicated times. Recombinant EphrinA5 (#374‐EA‐200, R&D Systems) was added to medium at the indicated concentrations for 48 h. The used concentrations of inhibitors were 10 µm Cpd1 (#sc‐314230; Santa Cruz Biotechnology), 0.5 µm crizotinib (#TM‐T1661‐1; TargetMol), and 10 µm dynasore (#SI‐D7693; Sigma‐Aldrich).

### T Cell Activation and Isolation In Vitro

T cells isolated and expanded from human PBMCs (STEMCELL Technologies) are prepared as described previously.^[^ ^43^ ^]^ Briefly, T cells were isolated from PBMCs by EasySep Human T Cell Isolation Kit (#17951; STEMCELL Technologies) and expanded by ImmunoCult‐XF T cell expansion medium (#10981; STEMCELL Technologies) for 10 d according to the manufacturer's protocol. To further activate T cells in vitro, human Jurkat or PBMC‐derived T cells combined with the treatment of phosphate‐buffered saline (PBS), RNase1, or CM from cancer cell lines as indicated times or concentrations were stimulated in 25 µL mL^−1^ of ImmunoCult Human CD3/CD28/CD2 T Cell Activator (#10990; STEMCELL Technologies), 10 ng mL^−1^ of human recombinant IL‐2 (#589104; Biolegend), and 1 µg mL^−1^ of phytohemagglutinin‐L (PHA‐L, #11249738001; Roche) for 2–48 h. For the enrichment of CD4^+^ and CD8^+^ T cells from PBMC‐derived T cells, PBMC‐derived T cells upon the stimulation of activation combined with the treatment of PBS, RNase1 (1 µg mL^−1^), or CM from MDA‐MB‐231 stable cell lines for 24 h were subject to CD4^+^ or CD8^+^ T cell isolation by using EasySep Human CD4+ T Cell Isolation Kit (#17952; STEMCELL Technologies) or EasySep Human CD8+ T Cell Isolation Kit (#17953; STEMCELL Technologies) according to the manufacturer's protocol.

### Plasmids, shRNA Clones, and Knocking Out Constructs

The information of pCDH‐CMV‐MCS‐EF1‐puro (Empty vector), pCDH‐R1 (Flag‐tagged RNase1), pCDH‐R1‐H12A (Flag‐tagged RNase1 with H12A mutation; Enzyme‐dead RNase1), pLentiCRISPRv2‐KO‐Ctrl (Control vector for knockout experiments), and pLentiCRISPRv2‐KO‐R1 for RNase1 gene knockout has been described in the previous study.^[^ ^6b^ ^]^ The pCDH‐R1‐5A (Flage‐tagged RNase1 with the substitute of 87‐TNGSR‐91 to 87‐AAAAA‐91) was generated by whole gene synthesis and sub‐cloned into pCDH‐CMV‐MCS‐EF1‐puro vector. The pCDH‐mR1 (mouse RNase1: NM_011271.2) and pCDH‐mR1‐H12A (mouse RNase1 with H12A mutation; Enzyme‐dead mouse RNase1) were produced through the inserts generated from whole gene synthesis and sub‐cloning the inserts into pCDH‐EF1‐MCS‐IRES‐puro/NEO vector. Chicken OVA was amplified by polymerase chain reaction (PCR) from pcDNA3‐OVA (#64599; Addgene) and sub‐cloned into pCDH‐EF1‐MCS‐IRES‐puro vector for the presentation of MHC class I specific epitope of OVA. For knockdown of human or mouse RNase1 by short hairpin RNAs, the shRNA vectors pLKO.1‐sh‐Ctrl (nontarget shRNA control; ASN0000000004 TRC1.Scramble; Target Sequence: CCTAAGGTTAAGTCGCCCTCG), pLKO.1‐sh‐R1#1 against RNase1 (TRCN0000373318; Target Sequence: TCCTTCTGCTTGTCCTGATAC), pLKO.1‐sh‐R1#2 against RNase1 (TRCN0000373374; Target Sequence: CTAAGGTCAGAGCAGCGAGAT), pLKO.1‐sh‐mR1#1 against mouse RNase1 (TRCN0000066943; Target Sequence: CCACTTTGATGCTACTGTGTA), pLKO.1‐sh‐mR1#2 against mouse RNase1 (TRCN0000066946; Target Sequence: CACCTACTGCAACCAAATGAT), pLKO.1‐sh‐ALK#1 against ALK (TRCN0000000787; Target Sequence: AGAAGAAGAAATCCGTGTGAA), pLKO.1‐sh‐ALK#2 against ALK (TRCN0000199879; Target Sequence: GCCCTGATCATCAGCAAATTC), pLKO.1‐sh‐EphA4#1 against EphA4 (TRCN0000196950; Target Sequence: GACTTGCAAGGAGACGTTTAA), and pLKO.1‐sh‐EphA4#2 against EphA4 (TRCN0000010165; Target Sequence: TCAGTCCGTGTGTTCTATAAA) were obtained from the RNA Technology Platform and Gene Manipulation Core Facility (RNAi core) of the National Core Facility for Biopharmaceuticals at Academia Sinica in Taiwan.

### Generation of Stable Cell Lines

According to the types of cell lines or vectors, different concentrations of antibiotics were applied for stable cell selection. 1 µg mL^−1^ of puromycin was used for selection in FaDu, SAS, MOC‐L1, and 4T1 stable cells; 1.5 µg mL^−1^ of puromycin in KPL4 and MDA‐MB‐231 stable cells; 1500 µg mL^−1^ of G418 in E0771 stable cells; 1.5 µg mL^−1^ of puromycin and 1500 µg mL^−1^ of G418 in OVA‐expressing E0771 and B16‐F10 stable cells. At least four weeks were spent for stable cell selection. Puromycin (#HY‐B1743A) and G418 (#HY‐17561) were purchased from MCE Corporation (Monmouth Junction, NJ, USA).

### Lentivirus Production and Transduction

To package lentivirus, pCMV delta 8.2, pDVsVg, and lentivirus‐based expression vectors were cotransfected into 293T cells by Lipofectamine LTX with Plus Reagent (#15338100; Life Technologies). The medium was replaced at 24 h after transfection. After 48 h, packaged viruses were harvested through centrifugation and filtered with a 0.45 µm filter. Cells (30% confluence) in six‐well plates were incubated in lentivirus‐containing medium with 10 µg mL^−1^ polybrene (#TR‐1003‐G; EMD Millipore) for 24 h. Infected cells were allowed to recover for 24 h and then selected by the appropriate antibiotics for at least 4 weeks.

### RNA Extraction, Reverse Transcription, and Quantitative RT‐PCR Analysis

Cell lysates were harvested using the TRIzol Reagent (#15596026; Thermo Fisher Scientific) and total RNA isolated with the RNeasy Mini Kit (#74104; Qiagen) according to the manufacturers’ protocols. cDNA was synthesized through the reverse transcription from purified RNA using SuperScript III First‐Strand cDNA synthesis system (#18080051; Thermo Fisher Scientific) according to the manufacturer's protocol. Quantitative RT‐PCR was performed using the CFX Connect Real‐Time PCR Detection System (Bio‐Rad). glyceraldehyde‐3‐phosphate dehydrogenase (GAPDH), β‐actin, or 18S was selected as an internal control for mRNA expression. All the results of quantitative RT‐PCR were performed by three independent experiments with two or three technical replicates. The data were analyzed by the 2^−ΔΔCT^ method. Primer sequences (5′–3′) were as follows:

IL‐2‐Forward: AGAACTCAAACCTCTGGAGGAAG, IL‐2‐Reverse: GCTGTCTCATCAGCATATTCACAC; IFNG‐Forward: GAGTGTGGAGACCATCAAGGAAG, IFNG‐Reverse: TGCTTTGCGTTGGACATTCAAGTC; IL‐6‐Forward: AGACAGCCACTCACCTCTTCAG, IL‐6‐Reverse: TTCTGCCAGTGCCTCTTTGCTG; GM‐CSF‐Forward: GGAGCATGTGAATGCCATCCAG, GM‐CSF‐Reverse: CTGGAGGTCAAACATTTCTGAGAT; hRNase1‐Forward: GTGATTGCAGAAACTGGCCT, hRNase1‐Reverse: CAGCACCAGCAGTATCAGGA; PD‐1‐Forward: AAGGCGCAGATCAAAGAGAGCC, PD‐1‐Reverse: CAACCACCAGGGTTTGGAACTG; LAG‐3‐Forward: GCAGTGTACTTCACAGAGCTGTC, LAG‐3‐Reverse: AAGCCAAAGGCTCCAGTCACCA; TIM‐3‐Forward: GACTCTAGCAGACAGTGGGATC, TIM‐3‐Reverse: GGTGGTAAGCATCCTTGGAAAGG; GAPDH‐Forward: AAGGTGAAGGTCGGAGTCAA, GAPDH‐Reverse: AATGAAGGGGTCATTGATGG; β‐actin‐Forward: CACCATTGGCAATGAGCGGTTC, β‐actin‐Reverse: AGGTCTTTGCGGATGTCCACGT; 18S‐Forward: CGGCGACGACCCATTCGAAC, 18S‐Reverse: GAATCGAACCCTGATTCCCCGT; mRNase1‐Forward: CAGCACAGAAGTTTCAGCGGCA, mRNase1‐Reverse: CTCATGCACGAAGGTGTTCACG; mGAPDH‐Forward: CGTCCCGTAGACAAAATGGT, mGAPDH‐Reverse: TTGATGGCAACAATCTCCA; mβ‐actin‐Forward: CATTGCTGACAGGATGCAGAAGG, mβ‐actin‐Reverse: TGCTGGAAGGTGGACAGTGAGG.

### Preparation of CM from Cell Culture

8 mL of serum‐free medium were used to culture cells. After 24 h, Cell debris was removed from CM containing secreted proteins by 0.45 µm filters. The volume of CM was concentrated to 300 µL by Amicon Ultra‐15 Centrifugal Filter Units (#UFC900324EMD; Millipore) at 5000 × *g*.

### Preparation of Samples for MS Analysis

For the MS result in CM of KPL4 cells, the SDS‐PAGE gel loaded with 5 µg of CM were subjected to electrophoresis. The desired protein bands were cut and applied to MS analysis by OmicsLab Co., Ltd. (New Taipei City, Taiwan). For the identification of putative RNase1‐associated proteins, cell lysates from activated Jurkat T cells treated with CM of 293T‐pCDH (Control vector) or R1 (Flag‐tagged RNase1 overexpression) were collected and applied to immunoprecipitation by antiflag antibody‐conjugated magnetic bead (#M8823; Sigma‐Aldrich). Total proteins from each sample after immunoprecipitation were loaded onto SDS‐PAGE for electrophoresis and then subjected to MS analysis by Novogene Co., Ltd. (Beijing, China).

### Analysis of Cancer Patient Tissues from Databases

All samples were available in TCGA data portal (https://tcga‐data.nci.nih.gov/tcga/). Correlation analysis between RNase1 expression versus exhausted T‐cell signatures (including HAVCR2, TIGIT, LAG3, PDCD1, CXCL13, LAYN), STAT1/ITGB2 expression versus effector T‐cell signatures (including CX3CR1, FGFBP2 and FCGR3A), or STAT1/ITGB2 expression versus RNase1‐related effector cytokine signatures (including IL‐2, IL‐6, IFN‐γ, and GM‐CSF) were performed using the GEPIA2 database with spearman correlation (http://gepia2.cancer‐pku.cn/#correlation). The gene expression profiles in cancer tissues compared to normal tissues were acquired from the TNMplot (https://tnmplot.com/analysis/) or GENT2 (http://gent2.appex.kr/gent2/) databases. RNA‐sequencing expression profiles and corresponding clinical information for the cancer types of HNSC, BRCA, LIHC, LUSC, COAD, and ESCA were downloaded from the TCGA dataset. Differential expression analysis and gene correlation analysis were employed by using the ggplot2 R package and pheatmap R package in R software (version 4.0.3), to identify significant associations between RNase1 expression and immune‐checkpoint‐relevant transcripts, namely CD274 (PD‐L1), CTLA4, HAVCR2 (TIM‐3), PDCD1 (PD‐1), LAG‐3, PDCD1LG2 (PD‐L2), TIGIT, and SIGLEC15. For GSEA, the BRCA, HNSC, and LIHC cohort from TCGA database were categorized into high and low expression groups on the basis of median values for RNase1 expression, then applied to the analysis using GSEA software (version 4.3.2; http://software.broadinstitute.org/gsea). The T cell exhaustion signatures (including HAVCR2, TIGIT, LAG3, PDCD1, CXCL13, LAYN) was used as the reference gene set.^[^ ^44^ ^]^ The number of permutations was set at 1000. The correlation between RNase1 expression and infiltrating immune cells for breast cancer and HNSC was carried out as previously described.^[^ ^11e^ ^]^

### Flow‐Cytometric Analysis

To determine the expression of PD‐1, LAG‐3, and TIM‐3 on T‐cell membrane, 1 × 10^6^ cells were collected and suspended in Cell Staining Buffer (#420201; BioLegend) and stained for 30 min with Zombie Aqua fixable viability kit (1:100, #423101; BioLegend), APC‐conjugated anti‐PD‐1 (1:50, #329907; BioLegend), phycoerythrin (PE)‐conjugated anti‐LAG‐3 antibody (1:50, #369205; BioLegend), fluorescein isothiocyanate (FITC)‐conjugated anti‐TIM‐3 antibody (1:50, #345021; BioLegend), and iFluor 647‐conjugated anti‐His tag antibody (1:50, #A01802‐100; Thermo Fisher) by using APC Mouse IgG1 (1:50, #400119; BioLegend), PE Mouse IgG1 (1:50, #400113; BioLegend), FITC Mouse IgG1 (1:50, #400109; BioLegend), and Alexa Fluor 647 Mouse IgG1 (1:50, #51‐4714‐81; Thermo Fisher) as staining controls. For detection of MHC class I specific epitope of OVA on cell membrane, cells were stained for 30 min with PE antimouse H‐2K^b^ bound to SIINFEKL Antibody (1:20, #141603; BioLegend) by using PE Mouse IgG1 (1:20, #400111; BioLegend) as a staining control. Stained cells were tested by CytoFLEX Flow Cytometer (Beckman Coulter, Indianapolis, IN, USA) and collected data were analyzed by the FlowJo software.

### Determination of Amounts of IL‐2 and INF‐γ in CM, or RNase1 Concentrations in Patient Serum by ELISA

The amounts of IL‐2 and INF‐γ in CM from cell culture were measured by LEGEND MAX Human IL2 ELISA Kit (#431807; BioLegend) and LEGEND MAX Human IFN‐r ELISA Kit (#430107; BioLegend), respectively. The concentration of RNase1 in patient serum was determined by Human RNASE1 ELISA Kit (#ELH‐RNASE1‐1; Raybiotech). All procedures are performed according to the manufacturer's instructions. In brief, serum was diluted 1000‐fold with PBS buffer. CM was diluted 10 or 120‐fold with PBS buffer for detection of IL‐2 or INF‐γ, respectively. Standards or diluted samples were loaded to each well of a plate and incubated at 37 °C for 2 h. Next, 100 µL biotin‐conjugated antibody was added to each well and then incubated at 37 °C for 1 h. After washing, 100 µL of a horseradish peroxidase conjugated Avidin was added to each well, and mixtures were incubated for 30 min at 37 °C. After washing, each well was added with 100 µL 3,3',5,5'‐Tetramethylbenzidine substrate solution for 20 min at 37 °C in the dark. Finally, the enzyme‐substrate reaction was stopped by 50 or 100 µL stop solution. Optical density (OD) value of each well was subsequently analyzed at a wavelength of 450 and 570 nm by a Synergy H1 Hybrid Multimode reader (BioTek Instruments). The concentration of targeted protein in samples was calculated by the comparison of the OD value of samples to the standard curve.

### Cell Counting Kit‐8 (CCK8) Proliferation Assay

The T‐Pro Cell Counting Kit (CCK‐8, #JK95‐C008S; T‐Pro Biotechnology) was used to evaluated the cell proliferation of CD4^+^ and CD8^+^ T cells isolated from activated PBMC‐derived T cells by RNase1 treatment. 5000 CD4^+^ or CD8^+^ T cells treated with or without 1 µg mL^−1^ RNase1 were seeded on a 96‐well plate. At each time point, 20 µL CCK8 solution was added to each well and incubated for 2 h. Then, relative proliferative state was determined at 450 nm by using a microplate reader at the indicated time points.

### Cell Apoptotic Assay

The percentage of apoptotic cells were tested by Annexin V Apoptosis Detection Kit eFluor 450 (#88‐8006‐74; eBioscience). CD4^+^ or CD8^+^ T cells were isolated from activated PBMC‐derived T cells treated with or without RNase1 (1 µg mL^−1^) for 48 h. 5×10^5^ cells were collected and diluted in binding buffer. Cells were stained by Annexin V at room temperature for 30 min. After washing with binding buffer, cells were collected and suspended in 195 µL binding buffer. Finally, 5 µL 7‐Aminoactinomycin D was added to cells which were analyzed with a CytoFLEX Flow Cytometer (Beckman Coulter).

### Cell Cycle Analysis

Cell cycle analysis was performed by using propidium iodide (PI)/RNase staining solution (#4087; Cell Signaling Technology) according to manufacturer's protocol. Briefly, 5 × 10^5^ cells were collected and fixed with 75% ethanol at −20 °C overnight. After washing with PBS, 1 mL PI/RNase staining solution was added to cells and then incubated for 15 min in the dark. Cells were applied to cell cycle analysis by using a CytoFLEX Flow Cytometer (Beckman Coulter).

RNase A treatment and PI costaining was done using a commercially available PI/RNase staining solution (Cell Signaling, Germany). Cell cycle profiles were acquired with an FACSCalibur flow cytometer.

### T Cell‐Mediated Cancer Cell Killing Assays

Activated T cells were prepared by treatment with ImmunoCult Human CD3/CD28/CD2 T Cell Activator (25 µL mL^−1^), human recombinant IL‐2 (10 ng mL^−1^), and PHA‐L (1 µg mL^−1^) for 24 h. Cancer cells were seeded to 24‐well plates overnight and then incubated for 24–72 h with activated T cells. The cancer cell amounts, cocultured time, and ratios between cancer cells and activated T cells were indicated in Figure Legends. At the end points of experiments, PBS was used to remove T cells and cell debris. Living cancer cells on plates were fixed and stained for 1 h using a 0.1% crystal violet solution, then quantified by a spectrometer at OD 570 nm.

For time‐course measurement of T cell‐meditated cancer cell killing effect, 2 × 10^3^ MDA‐MB‐231 stable cells were stained by Incucyte Nuclight Rapid Red dye (1:500, #4717; Sartorius) and cultured in a 96‐well plate overnight. Then cancer cells containing Incucyte Caspase‐3/7 Green dye (1:1000, #4440; Sartorius) were cocultured with CD8^+^ T cells isolated from activated PBMC‐derived T cells at a 1:5 cell ratio. The signals of green and red fluorescent were measured and captured every 4 h in the Incucyte live‐cell analysis system (Essen Bioscience). Data analysis was carried out by using the integrated software.

For evaluate OT‐1 T‐cell cytotoxicity against cancer cells expressing MHC class I specific epitope of OVA, splenocytes were isolated from the spleen of OT‐I mice by rupturing the spleen in complete RPMI medium (RPMI medium supplemented with 10% fetal bovine serum, 50 µm 2‐mercaptoethanol, and 10 mm 4‐(2‐Hydroxyethyl)piperazine‐1‐ethanesulfonic acid). The isolated splenocytes were maintained in complete RPMI medium supplemented with 10 ng mL^−1^ of recombinant murine IL‐2 (#21212; PeproTech) and 0.75 µg mL^−1^ OVA SIINFEKL peptide (#vac‐sin; Invivogen) for 4 d of T cell expansion. For cancer cell killing assay, 2 × 10^4^ cancer cells were adhered to 24‐well plates overnight and then cocultured with expanded cytotoxic OT‐I T cells for 24 h. The effector‐to‐target (E:T) cell ratio was 1:1. After coculture for 24 h, T cells were removed and cancer cells were cultured for further 1 d. Finally, cancer cells on plates were fixed and stained for 1 h using a 0.1% crystal violet solution, then quantified by a spectrometer at OD 570 nm.

### In Vivo Murine Experiments

The indicated number of cancer cells were suspended in 50 µL of RPMI 1640 medium (#SH30255.02; Hyclone) or Dulbecco's modified Eagle's medium (#SH30243.02; Hyclone) mixed with 50 µL of the Matrigel (#354 234; Corning). The cancer cells were orthotopically injected into the mammary fat pad of mice for 4T1, E0771, and MDA‐MB‐231 cell lines or subcutaneously injected into the flanks of mice for MOC‐L1 cell line. After inoculation of tumor cells, tumor growth was measured twice per week for the duration shown in the figures and tumor volume was calculated using the formula 1/2 × length × width^2^. For the depletions of CD4^+^ or CD8^+^ T cells in the 4T1 mouse model, the experimental procedures were performed as previously described.^[^ ^45^ ^]^ Mice bearing tumors were treated with 200 µg mouse IgG1 isotype control (#BE0083; BioXCell), antimouse CD4 (#BE0003‐1; BioXCell), or antimouse CD8α (#BE0061; BioXCell) antibodies on day 3, 6, 9, 12, and 15 through intraperitoneal injections. To reconstitute human CD8^+^ T cells in the Prkdc^scid^Il2rg^null^ (NPG) mouse model, we performed and modified experiments as previously described.^[^ ^46^ ^]^ NPG mice were received 1×10^7^ CD8^+^ T cells intraperitoneally on Day 7 after tumor inoculation. CD8^+^ T cells were isolated from PBMC‐derived T cells upon activation for 24 h. To measure serum levels of RNase1 in BALB/c mice implanted with 4T1 cells, Mice were sacrificed on day 20 after tumor inoculation and their blood was collected. Serum was separated from blood by centrifugation at 3000 rpm for 10 min in a BD Microtainer (#365967; BD). The concentration of RNase1 in serum was determined by the mouse RNASE1 (Ribonuclease A) ELISA kit (#MBS8804573; MyBioSource) according to the manufacturer's instruction. NOD SCID, BALB/c, C57BL/6, and NPG mice (5‐week‐old females) were obtained from BioLASCO Taiwan Co., Ltd. (Taipei, Taiwan) or National Laboratory Animal Center of Taiwan (Taipei, Taiwan).

### CD8^+^ and CD4^+^ T‐Cell Profile Analysis by Fluorescence‐Activated Cell Sorting

The excised tumors were digested into single cells by using Mouse Tumor Dissociation Kit (#130‐096‐730, Miltenyl Biotec) and gentle MACS Octo Dissociator (#130‐096‐427, Miltenyl Biotec). After removal of red blood cells and blocking with antimouse CD16/CD32 antibody (#101302; BioLegend, 1:50), 1 × 10^6^ single cells were suspended in Cell Staining Buffer (#420201; BioLegend) and stained for 30 min with PE‐CD45 antibody (#103105; BioLegend, 1:100), APC‐CD3ε antibody (#100311; 1:100; BioLegend), APC/Cy7‐CD8a antibody (#100713; BioLegend, 1:100), and Alexa Fluor 700‐CD4 Antibody (#100430; BioLegend, 1:50). To stain intracellular granzyme B and IFN‐γ, cells were fixed and permeabilized by Intracellular Staining Permeabilization Wash Buffer (#421002; Biolegend), following to staining with Pacific Blue‐IFN‐γ antibody (#505817; Bio‐Legend, 1:50), and FITC‐granzyme B antibody (#515403; BioLegend, 1:50). The VersaComp Antibody Capture Bead kit (#B22804; Beckman Coulter) was applied to generate a compensation matrix for multicolor applications of flow cytometry. Stained cells were analyzed using a CytoFLEX Flow Cytometer (Beckman Coulter). Data were processed by using the FlowJo software.

### RNase Enzymatic Activity Assay

RNase enzymatic activity was measured by Ambion RNaseAlert Lab Test kit (#AM1964; Thermo Fisher Scientific,) according to the manufacturer's protocol with minor modifications. In brief, a tube containing the fluorescent substrate was loaded with 5 µL of tenfold RNaseAlert buffer, and then mixed with 45 µL CM. The mixture was sequentially transferred to a well of 96‐well plate. The real‐time fluorescence signaling was detected at 1 min intervals for 1 h through a Synergy H1 Hybrid Multimode reader (BioTek Instruments).

### Western Blotting and Coimmunoprecipitation (co‐IP)

To perform western blotting, cells were collected and lysed in the Cell Lysis Buffer (#9803; Cell Signaling Technology) supplemented with protease inhibitor cocktail (#HY‐K0011, MedChemExpress). Protein concentration was determined by Pierce BCA Protein Assay kit (#23227; Thermo Fisher Scientific). Immunoblotting was carried out with primary antibodies against human RNase1 (#HPA001140; Sigma Aldrich, 1:1000), STAT1 (#9172; Cell Signaling Technology, 1:1000), pTyr701‐STAT1 (#9167; Cell Signaling Technology, 1:1000), pSer727‐STAT1 (#8826; Cell Signaling Technology, 1:1000), mouse RNase1 (#E‐AB‐19132; Elabscience, 1:1000), alpha Tubulin (#11224‐1‐AP; Proteintech, 1:3000), Histone H3 (#GTX122148; GeneTex, 1:3000), Sodium Potassium ATPase (#ab76020; Abcam, 1:6000), and β‐actin (NB600‐501; Novus, 1:10000). The signals of antibody binding were detected using T‐Pro LumiDura Chemiluminescence Detection kit (#JT96‐K006 M; T‐Pro Biotechnology, New Taipei City, Taiwan) on a ChemiDoc Imaging System (Bio‐Rad). The quantitative data of western blotting were analyzed using ImageJ software (National Institutes of Health, Maryland, USA). For co‐IP assays, cells were harvested and lysed by Pierce IP Lysis Buffer (#87787; Thermo Fisher Scientific) including protease inhibitor cocktail (#HY‐K0011, MedChemExpress). 20 µL of anti‐FLAG M2 magnetic beads (#M8823, Merck) was equilibrated with PBS and mixed with 700 µg of cell lysates at 4 °C for overnight. The magnetic beads bound with target proteins were washed with PBS and eluted with 2X Laemmli sample buffer. For protein samples after co‐IP applied to western blotting, the horseradish peroxidase‐conjugated antimouse IgG for IP (#ab131368; Abcam) or VeriBlot for IP Detection reagent (#ab131366; Abcam) was used as secondary antibody in the process of western blotting.

### Proposed a Binding Model for RNase1 and STAT1

The atomic structures of RNase1 (PDBID: 1Z7X) and STAT1 (PDBID: 1YVL) were obtained from Protein Data Bank (https://www.rcsb.org/), and applied to HDOCK server (http://hdock.phys.hust.edu.cn/)^[^ ^28^ ^]^ to generate the binding models. The quality of resulting models was assessed through docking and confidence scores, which are shown in the HDOCK results. According to the definition of the HDOCK server, a good binding model should have a more negative docking score, generally lower than ‐200, and the confidence score should be higher than 0.7. The docking and confidence scores of the top 5 RNase1/STAT1 models were shown in Figure 5J. The model with highest scores was used for subsequent analysis.

### Cell Fractionation

To separate protein fractions of cytoplasm, nucleus, and membrane from activated PBMC‐derived T cells treated with or without RNase1 for 48 h, the Fraction‐PREP Cell Fractionation Kit (#ab288085; Abcam) was used. The procedures were carried out according to the manufacturer's instruction. 20 µg protein of each fraction was applied to western blotting analysis to validate the success of cell fractionation. Histone H3, Sodium Potassium ATPase, and α‐tubulin served as positive controls for nuclear, membrane, and cytosolic proteins, respectively.

### ICC Staining

4 × 10^4^ activated PBMC‐derived T cells treated without or with RNase1 (1 µg mL^−1^) for 48 h were placed onto polysine adhesion microscope slides (#J2800AMNZ; Epredia) by using a cytospin centrifuge. Cells were fixed in 4% paraformadehyde for 10 min at RT, permeabilized by the treatment of 0.3% Triton X‐100 for 10 min and then incubated in the blocking buffer (PBS including 10% donkey serum, 1% BSA, 0.01% Tween20) for 1 h at RT. Samples were stained with unconjugated or conjugated primary antibodies against RNase1 (#HPA001140; Sigma‐Aldrich, 1:50), STAT1 (#AHO0832; Thermo Fisher Scientific, 1:250), EphA4 (#PA5‐32754; Thermo Fisher Scientific, 1:200), ALK(#MA5‐14528; Thermo Fisher Scientific, 1:50), and His‐tag (conjugated with Alexa Fluor 647, #MA1‐135‐A647; Thermo Fisher Scientific, 1:50) in antibody dilution buffer (PBS including 1% BSA and 0.01% Tween20) overnight at 4 °C, followed by Alexa Fluor 488‐goat antirabbit IgG (#A‐11034; Thermo Fisher Scientific, 1:1000) or Alexa Fluor 594‐donkey antimouse IgG (#A‐21203; Thermo Fisher Scientific, 1:1000) secondary antibodies at RT for 1 h. The samples were covered by coverslips in mounting medium with DAPI (#ab104139; Abcam). Three random images were captured for each sample by using the Nikon Ti2‐E AXR confocal microscope. The colocalization quantitative analysis of RNase1 and STAT1 in activated PBMC‐derived T cells treated with RNase1 was calculated using Pearson's correlation coefficient and Manders' overlap coefficients with the ImageJ plugin Coloc 2. For Pearson's correlation coefficient, the value range is from −1.0 to 1.0, where 0 indicates no significant correlation and −1.0 shows a complete negative correlation. A positive number indicates the degree of overlap of fluorescent signals. For Manders' overlap coefficients, values are between 0 and 1.0, with 1.0 indicating perfect colocalization for both fluorescent signals and zero meaning no overlap.

### Statistical Analysis

Data are shown as mean ± standard deviation (SD). To compare the continuous variables between groups, the two‐sided Unpaired Student's *t*‐test or ANOVA test was used as indicated in Figure Legends. Unless indicated otherwise by the figure legends, statistical analyses were typically conducted with the control groups as the first groups in the panels. Statistical data were analyzed using Prism 8 software (GraphPad Software 8) or the R software (version 4.0.3). Sample size for each statistical analysis was indicated in the figure legends. Statistical significance was evaluated by the *P*‐value of the statistical test assessments. A *P*‐value was considered as significant (*p < 0.05), strongly significant (**p < 0.01), or highly significant (***p < 0.001).

## Conflict of Interest

The authors declare no conflict of interest.

## Author Contributions

W.‐H.Y. and X.Y. designed and performed the experiments, analyzed data, and wrote the paper. B.‐Y.H., H.‐Y.R., and B.C. performed experiments and analyzed data. P.Y. analyzed and interpreted patient data from the TCGA database. H.‐C.W. analyzed and interpreted the protein docking model. C.‐H.C. analyzed and interpreted the colocalization quantitative analysis. H.‐H.W., H.‐R.Y., and S.‐C.W. provided the OT‐1 mouse model and scientific input. J.‐H.C. provided scientific input and review the paper. M.‐HY. provided and analyzed serum of head and neck cancer patients. M.‐C.H., M.‐H.Y., X.Y., and W.‐H.Y. supervised the entire project, designed the experiments, and wrote the paper.

## Supporting information



Supporting Information

## Data Availability

The data that support the findings of this study are available from the corresponding author upon reasonable request.
