# Supplementary material for: Ribonuclease 1 Induces T‐Cell Dysfunction and Impairs CD8+ T‐Cell Cytotoxicity to Benefit Tumor Growth through Hijacking STAT1
Source: Adv Sci (Weinh). 2025 Feb 11;12(13):2404961. doi: 10.1002/advs.202404961 (PMC11967817; doi:10.1002/advs.202404961)
Supplement: Supplementary file 1 — Supporting Information [file ADVS-12-2404961-s001.docx]

**Supporting Information**

**
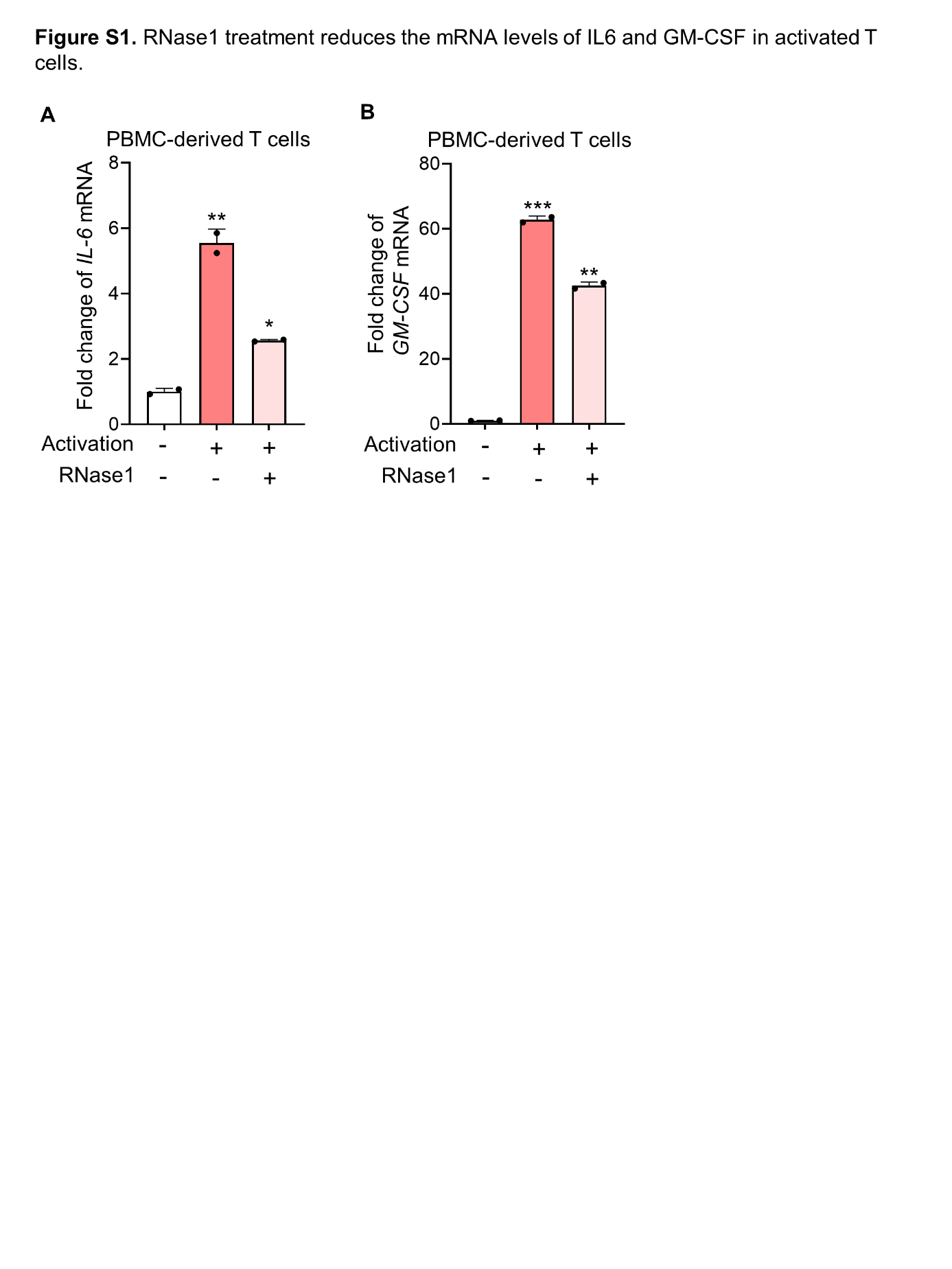
**

**Figure S1.** RNase1 treatment reduces the mRNA levels of IL-6 and GM-CSF in activated T cells. Quantitative RT-PCR analysis of (A) *IL-6* and (B) *GM-CSF* mRNA expression in unactivated PBMC-derived T cells and activated PBMC-derived T cells treated without or with 1 μg/ml recombinant RNase1 for 48 hrs. Three independent experiments with two technical replicates were carried out. Data are presented as mean ± SD, **P*, 0.01~0.05, ***P*, 0.001~0.01, and ****P*, < 0.001 by two-sided Unpaired Student’s *t*-test.

**
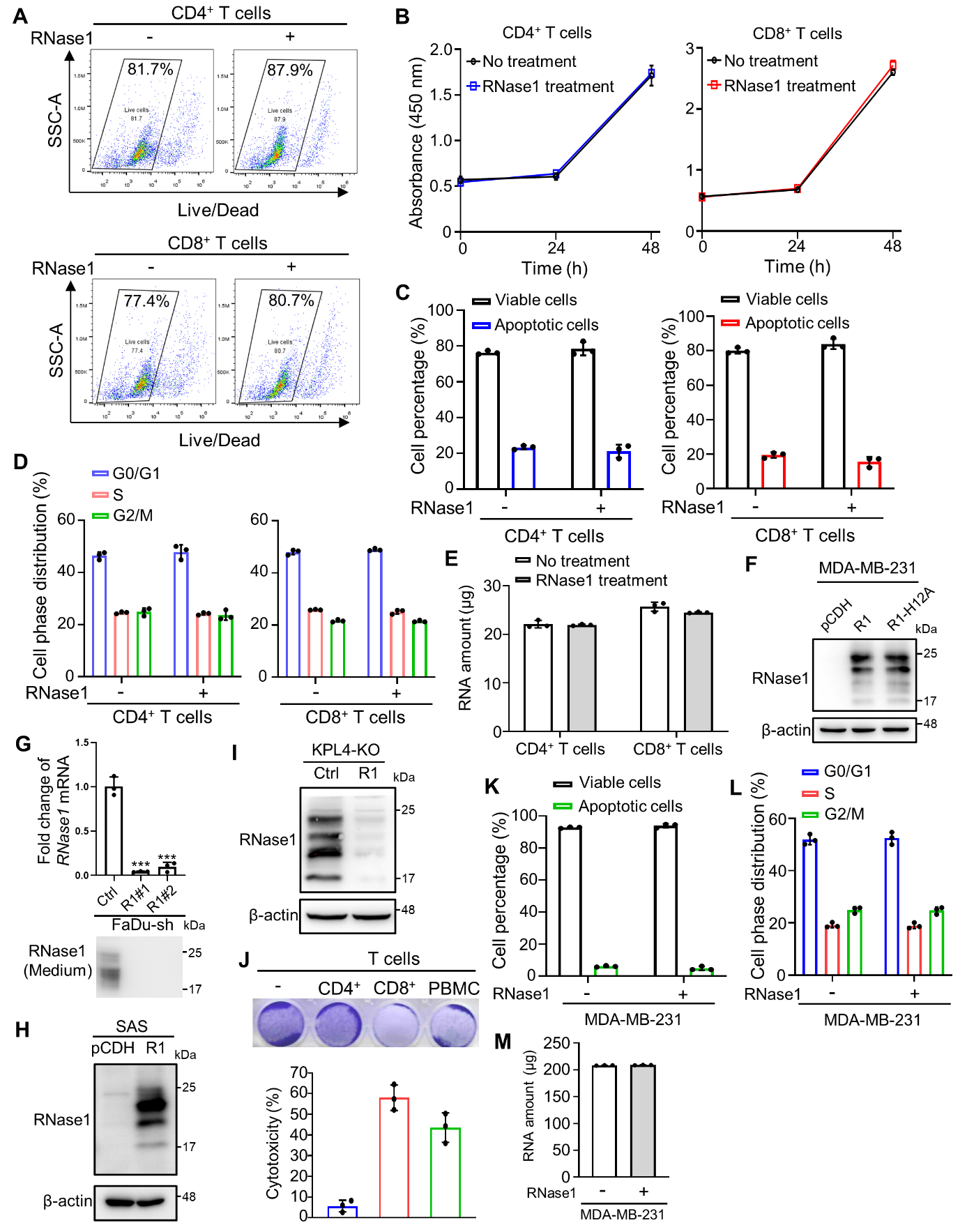
**

**Figure S2.** Treatment with 1 ug/ml RNase1 has no significant effect on cell proliferation, apoptosis, cell cycle, and total RNA amount of CD4^+^ T cells, CD8^+^ T cells, and cancer cells. A) Flow cytometric analysis of live cell percentage in CD4^+^ and CD8^+^ T cells isolated from activated PBMC-derived T cells treated with or without 1 μg/ml recombinant RNase1 for 24 hrs. Representative data from three independent experiments. B) Cell proliferation analysis of CD4^+^ and CD8^+^ T cells isolated from activated PBMC-derived T cells treated with or without 1 μg/ml recombinant RNase1 for 48 hrs by CCK8 assay. Representative data from two independent experiments (each experiment contains three technical replicates). C) Apoptosis analysis using flow cytometry after annexin V staining in CD4^+^ and CD8^+^ T cells isolated from activated PBMC-derived T cells treated with or without 1 μg/ml recombinant RNase1 for 48 hrs. Representative data from two independent experiments (each experiment contains three technical replicates). D) Cell cycle analysis using flow cytometry after propidium iodide (PI)/RNase staining in CD4^+^ and CD8^+^ T cells isolated from activated PBMC-derived T cells treated with or without 1 μg/ml recombinant RNase1 for 48 hrs. Quantitative data from three independent samples. E) The evaluations of total RNA amount in CD4^+^ and CD8^+^ T cells (5 x 10^5^ cells) isolated from activated PBMC-derived T cells treated with or without 1 μg/ml recombinant RNase1 for 48 hrs. Quantitative data from three independent samples. F) Western blot analysis of cell lysates from MDA-MB-231-pCDH, R1, and R1-H12A cells with RNase1 and β-actin antibodies. Representative results from three independent experiments. G) Upper: Quantitative RT-PCR analysis of *RNase1* mRNA expression in FaDu-sh-R1#1 and #2 cells compared to FaDu-sh-Ctrl cells. Lower: Immunoblot of secreted RNase1 in CM collected from FaDu-sh-Ctrl, sh-R1#1, and sh-R1#2 cells. Representative data from three independent experiments. H) Western blot analysis of cell lysates from SAS stable clones expressing empty control (pCDH), and R1 with RNase1 and β-actin antibodies. Representative data from three independent experiments. I) Western blot analysis of cell lysates from KPL4-KO-Ctrl, and KO-R1 cells with RNase1 and β-actin antibodies. Representative data from three independent experiments. J) Representative images and quantitative results of T cell-mediated cancer cell killing assay. MDA-MB-231 cells (2 x 10^4^ cells) co-cultured with activated PBMC-derived T cells (PBMC), or CD4^+^ T cells (CD4^+^) and CD8^+^ T cells (CD8^+^) isolated from activated PBMC-derived T cells for 24 hrs were subjected to crystal violet staining to evaluate T-cell cytotoxicity. MDA-MB-231 to T-cell ratio, 1:10. Three independent experiments with three technical replicates were carried out. K) Apoptosis analysis using flow cytometry after annexin V staining in MDA-MB-231 cells treated with or without 1 μg/ml recombinant RNase1 for 48 hrs. Representative data from two independent experiments (each experiment contains three technical replicates). L) Cell cycle analysis using flow cytometry after propidium iodide (PI)/RNase staining in MDA-MB-231 cells treated with or without 1 μg/ml recombinant RNase1 for 48 hrs. Quantitative data from three independent samples. M) The evaluations of total RNA amount in MDA-MB-231 cells (5 x 10^6^ cells) treated with or without 1 μg/ml recombinant RNase1 for 48 hrs. Quantitative data from three independent samples. Data are presented as mean ± SD (C-E, G, J-M).

**
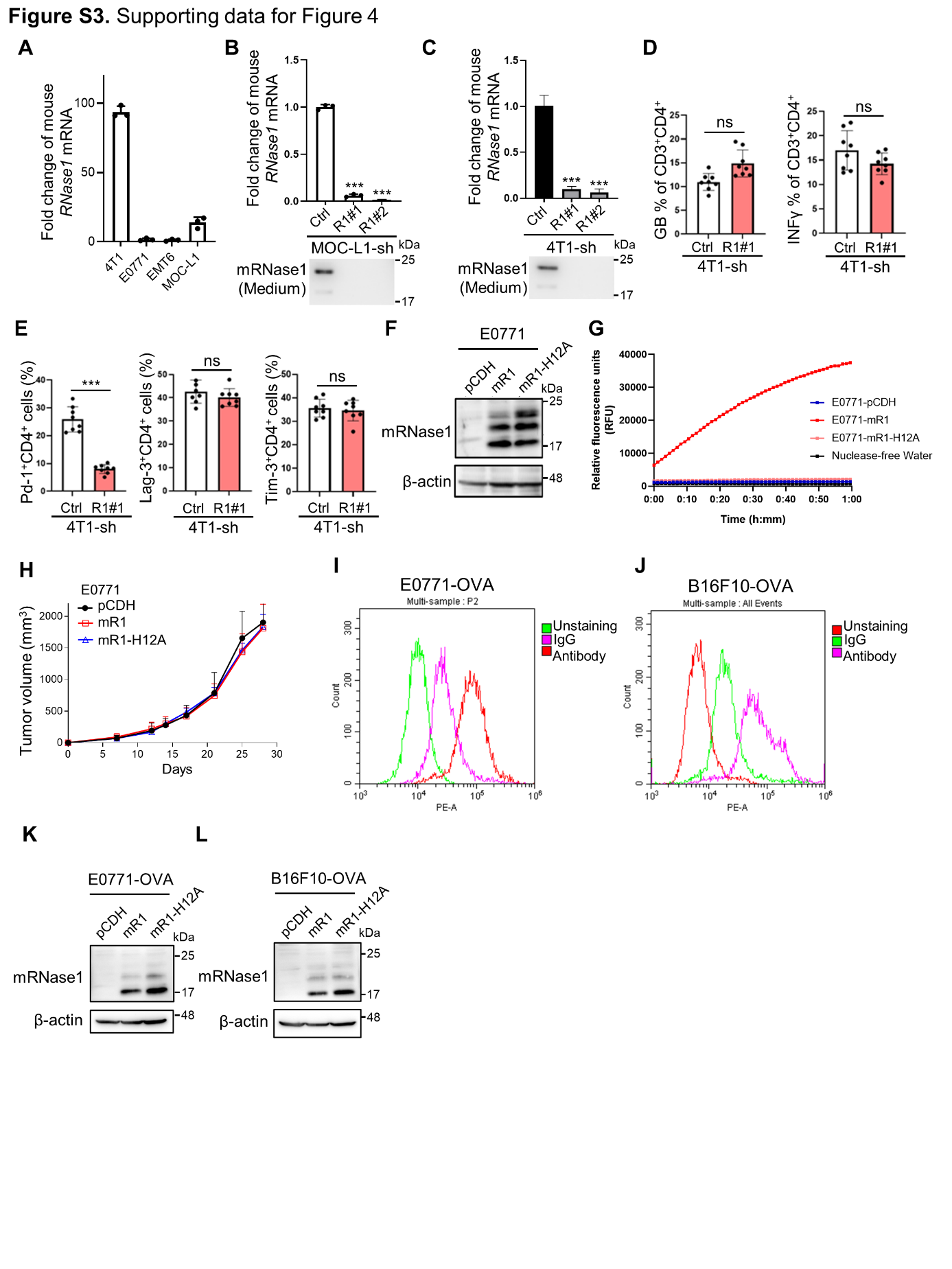
**

**Figure S3.** Supporting data for Figure 4. A) Quantitative RT-PCR analysis of mouse *RNase1* mRNA expression in 4T1, E0771, EMT6, and MOC-L1 cells. Representative data from three independent experiments. B) Upper: Quantitative RT-PCR analysis of mouse *RNase1* mRNA expression in MOC-L1-sh-R1#1 and #2 cells compared to MOC-L1-sh-Ctrl cells. Lower: Immunoblot of secreted RNase1 in CM collected from MOC-L1-sh-Ctrl, sh-R1#1, and sh-R1#2 cells. Representative data from three independent experiments. C) Upper: Quantitative RT-PCR analysis of mouse *RNase1* mRNA expression in 4T1-sh-R1#1 and #2 cells compared to 4T1-sh-Ctrl cells. Lower: Immunoblot of secreted RNase1 in CM collected from 4T1-sh-Ctrl, sh-R1#1, and sh-R1#2 cells. Representative data from three independent experiments. D) The percentage of CD3^+^CD4^+^ T cells-expressing granzyme B (GB) or IFNγ in 4T1-Ctrl and sh-R1#1 tumor tissues from mice according to flow cytometry analysis (n = 8 independent tissue samples). E) The percentage of CD3^+^CD4^+^ T cells-expressing Pd-1, Lag-3, or Tim-3 in 4T1-Ctrl and sh-R1#1 tumor tissues from mice according to flow cytometry analysis (n = 8 independent tissue samples). F) Western blot analysis of cell lysates from E0771-pCDH, mR1, and mR1-H12A cells with RNase1 and β-actin antibodies. Representative data from three independent experiments. G) Analysis of ribonucleolytic activity of CM collected from E0771-pCDH, mR1, mR1-H12A cells. Representative data from three independent experiments. H) E0771-pCDH, mR1, and mR1-H12A cells (2 x 10^4^) were orthotopically injected into NOD SCID mice (n = 10 mice per group). The tumor volume was measured. I) Flow cytometric analysis of MHC class I specific epitope of OVA on E0771-OVA cells. Cells without antibody staining or with immunoglobulin G (IgG) staining were used as control groups. Representative data from three independent experiments. J) Flow cytometric analysis of MHC class I specific epitope of OVA on B16F10-OVA cells. Cells without antibody staining or with immunoglobulin G (IgG) staining were used as control groups. Representative data from three independent experiments. K) Western blot analysis of cell lysates from E0771-OVA-pCDH, mR1, and mR1-H12A cells with RNase1 and β-actin antibodies. Representative data from three independent experiments. L) Western blot analysis of cell lysates from B16F10-OVA-pCDH, mR1, and mR1-H12A cells with RNase1 and β-actin antibodies. Representative data from three independent experiments. Data are presented as mean ± SD, ****P*, < 0.001, and NS, not significant by two-sided Unpaired Student’s *t*-test (B-E).


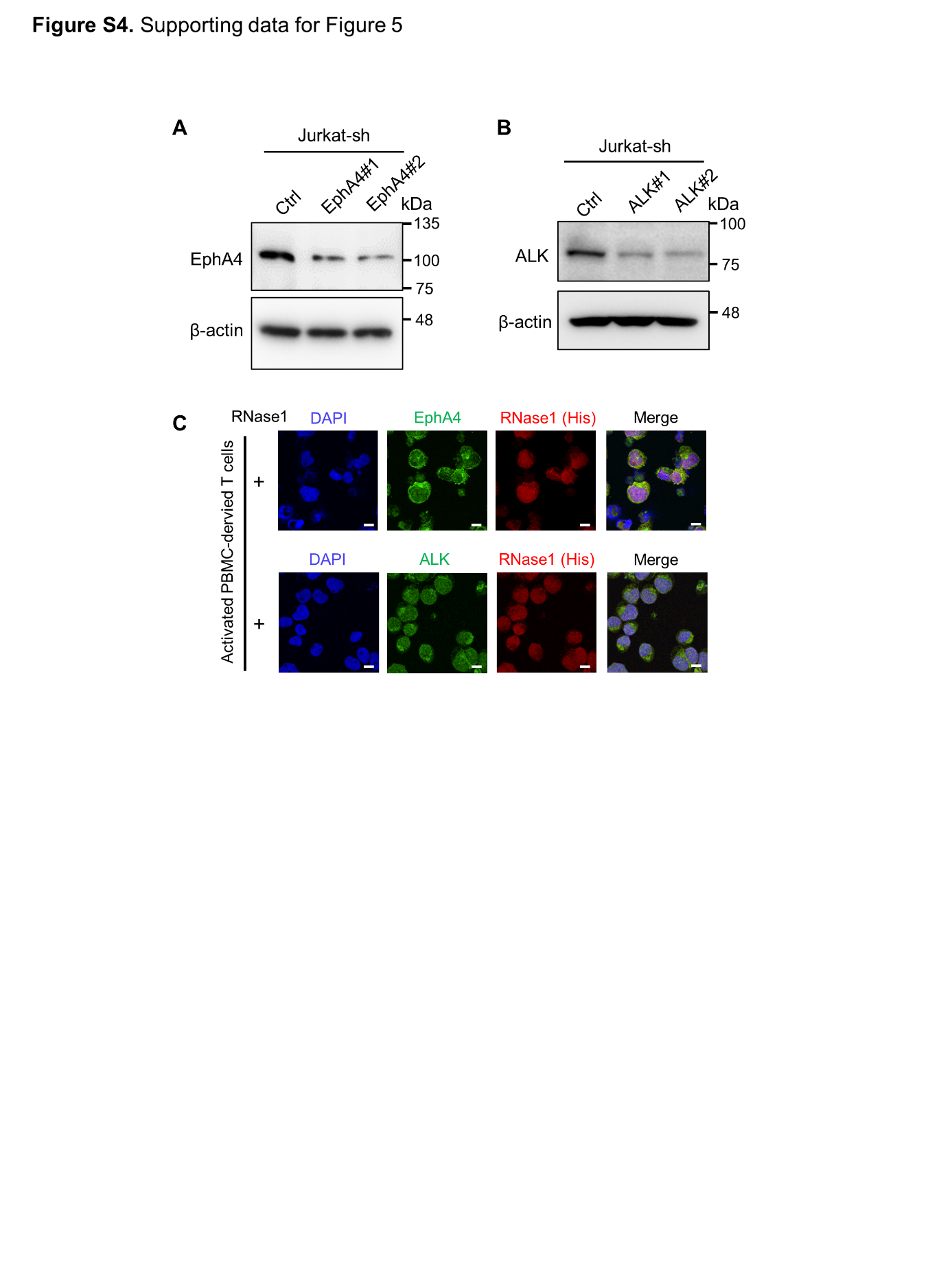


**Figure S4.** Supporting data for Figure 5. A) Western blot analysis of cell lysates from Jurkat-sh-Ctrl, EphA4#1, and #2 cells with EphA4 and β-actin antibodies. Representative data from three independent experiments. B) Western blot analysis of cell lysates from Jurkat-sh-Ctrl, ALK#1, and #2 cells with ALK and β-actin antibodies. Representative data from three independent experiments. C) Immunocytochemistry staining of RNase1 (His-tag), EphA4, and ALK in activated PBMC-derived T cells treated with RNase1 (1 μg/ml) for 48 hrs. Nuclei were counterstained with DAPI. Representative images of n=2 independent replicates. Scale bar: 10 μm.


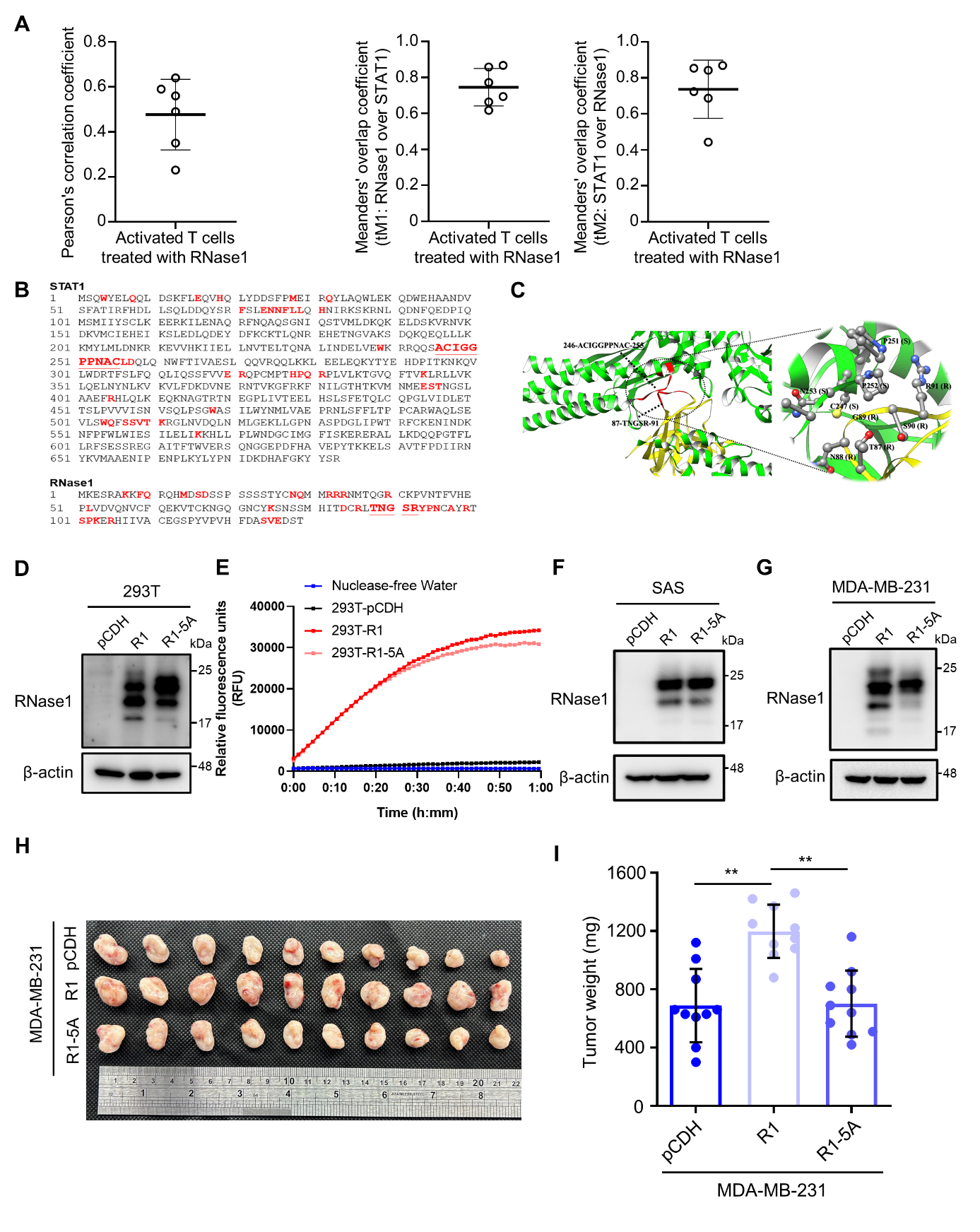


**Figure S5.** Supporting data for Figure 6. A) Quantification of the colocalization of RNase1 and STAT1 was performed using Pearson's correlation coefficient (left panel) and Manders' overlap coefficients tM1 and tM2 (right panels) in activated PBMC-derived T cells treated with RNase1. n = 6 images from two independent replicates. B) Residues located in the interacting interface on the RNase1/STAT1 binding model highest scores. These resides were colored in red. C) The 87-TNGSR-91 loop region on RNase1 and the adjacent loop region 246-ACIGGPPNAC-255 on STAT1. These two loops were highlighted in red. D) Western blot analysis of RNase1 of 293T-pCDH, R1, and R1-5A cells. β-actin served as a loading control. Representative data from three independent experiments. E) Analysis of ribonucleolytic activity of CM collected from 293T-pCDH, R1, R1-5A cells. Representative data from three independent experiments. F) Western blot analysis of cell lysates from SAS stable clones expressing empty control (pCDH), R1, and R1-5A with RNase1 and β-actin antibodies. Representative data from three independent experiments. G) Western blot analysis of cell lysates from MDA-MB-231 stable clones expressing empty control (pCDH), R1, and R1-5A with RNase1 and β-actin antibodies. Representative data from three independent experiments. H) The tumor volume image on day 21 in Figure 6(O). I) Quantitative analysis of tumor weight on day 21 in Figure 6(O). n = 10 independent samples. Data are presented as mean ± SD, **P*, 0.01~0.05, ***P*, 0.001~0.01, and ****P*, < 0.001 by two-sided Unpaired Student’s *t*-test.


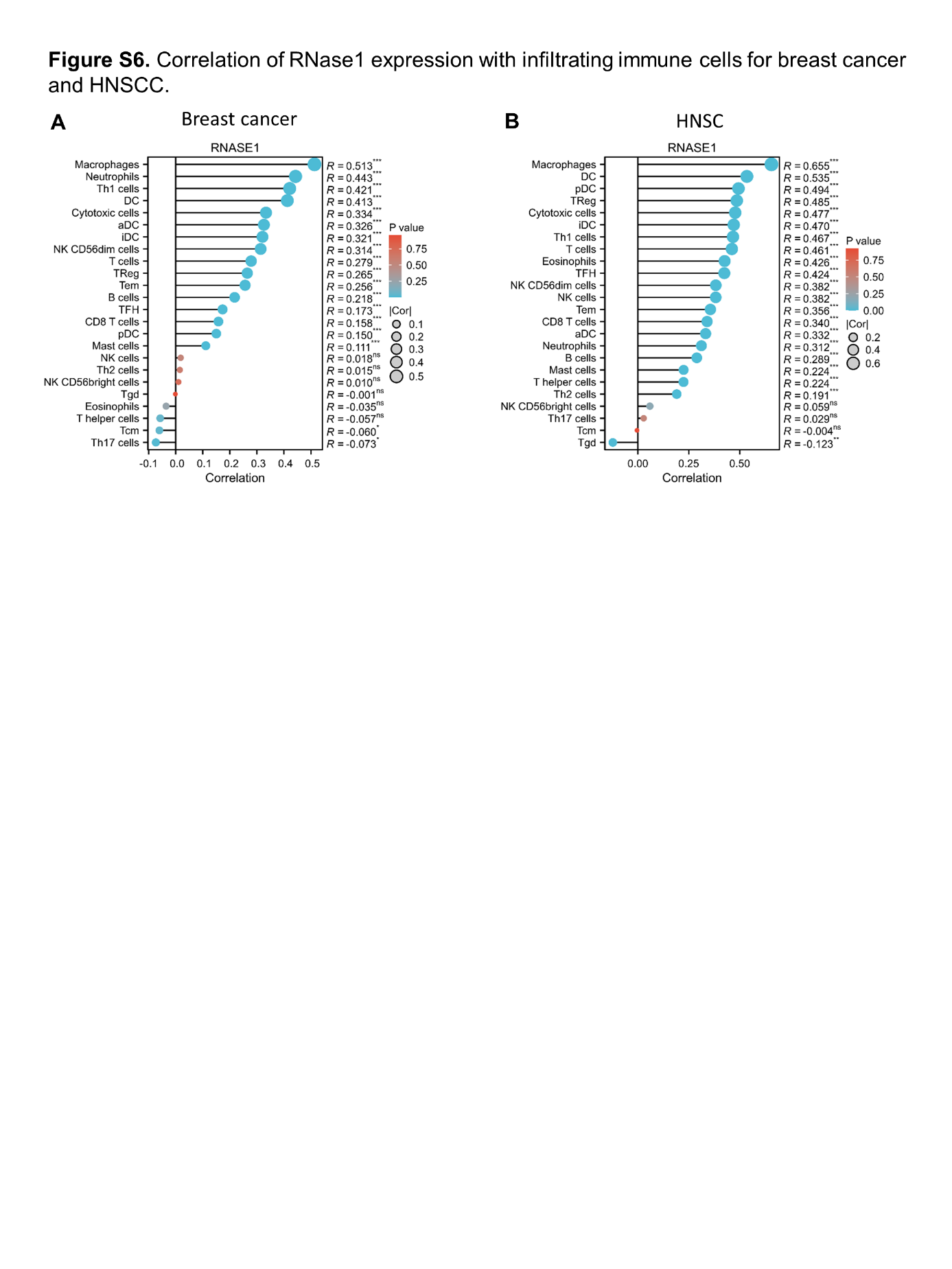


**Figure S6.** Correlation of RNase1 expression with infiltrating immune cells for breast cancer (A) and HNSCC (B). n = 1113 for breast cancer. n = 504 for HNSCC. Each dot's size indicates the correlation strength between RNase1 expression and immune cells. The *P* value is represented by the color of each dot.


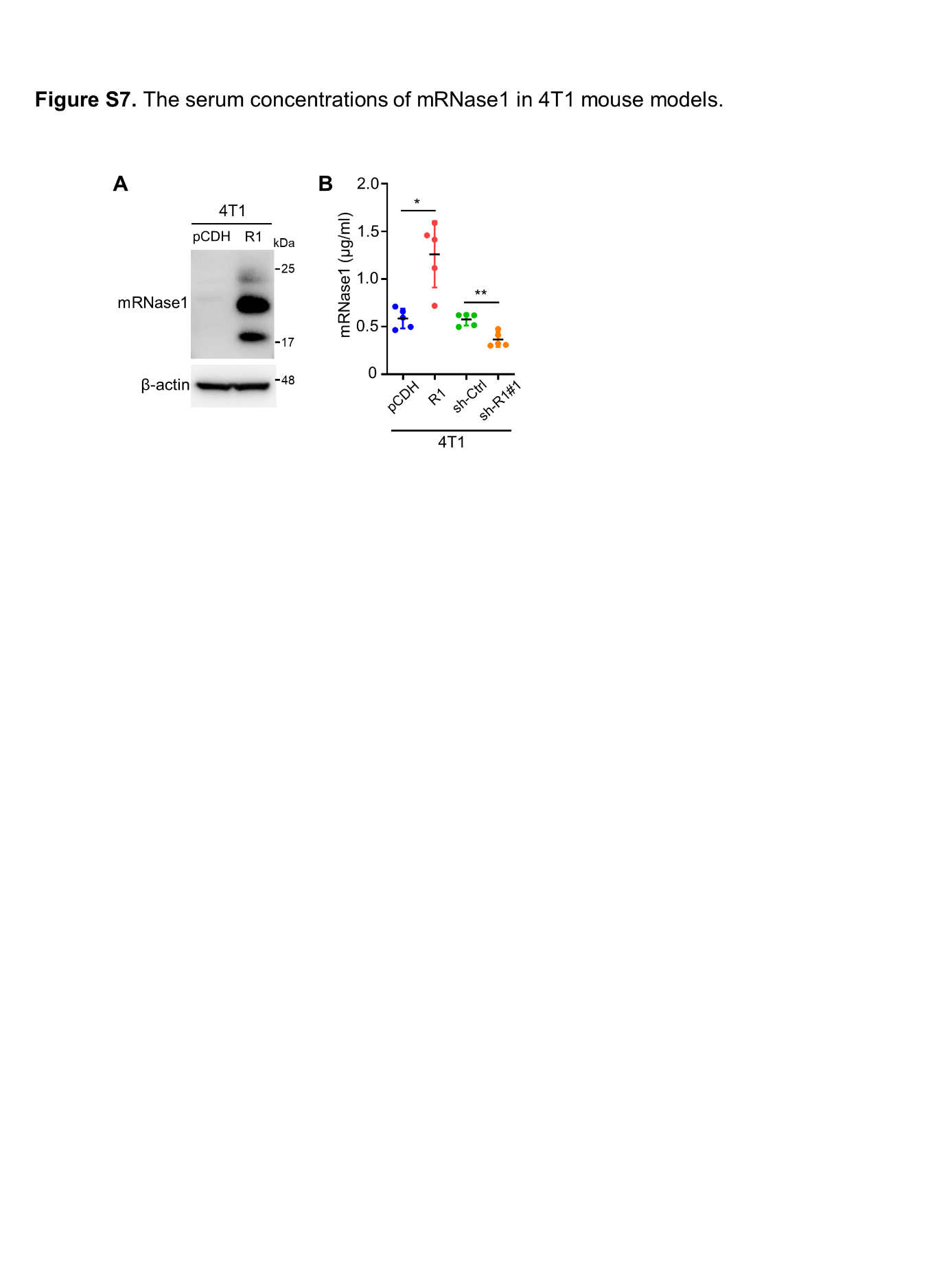


**Figure S7.** The serum concentrations of mRNase1 in 4T1 mouse models. A) Western blot analysis of mRNase1 of 4T1-pCDH and R1 cells. β-actin served as a loading control. Representative data from three independent experiments. B) The serum levels of mRNase1 in BALB/c mice bearing 4T1-pCDH, R1, sh-Ctrl, or sh-mR1#1 tumors (n = 5 mice per group) on day 20 after tumor inoculation.

**Supplementary Table S1.** Correlation of RNase1 expression with infiltrating immune cells for breast cancer

| **Immune cells** | **Correlation coefficient (R)** | ***P* value** |
| --- | --- | --- |
| Macrophages | 0.512894753 | 1.0619E-75 |
| Neutrophils | 0.442916292 | 1.11775E-54 |
| Th1 cells | 0.421292547 | 4.11434E-49 |
| DC | 0.412534385 | 5.69974E-47 |
| Cytotoxic cells | 0.333717197 | 2.33247E-30 |
| aDC | 0.325971417 | 5.76918E-29 |
| iDC | 0.320743642 | 4.77429E-28 |
| NK CD56dim cells | 0.313935378 | 7.03523E-27 |
| T cells | 0.278830432 | 2.53224E-21 |
| TReg | 0.264534611 | 2.81386E-19 |
| Tem | 0.25642466 | 3.59726E-18 |
| B cells | 0.217770892 | 2.05201E-13 |
| TFH | 0.173221291 | 6.01003E-09 |
| CD8 T cells | 0.157909678 | 1.18714E-07 |
| pDC | 0.150095623 | 4.89626E-07 |
| Mast cells | 0.11119598 | 0.000201566 |
| NK cells | 0.018388908 | 0.53997761 |
| Th2 cells | 0.015357642 | 0.608783426 |
| NK CD56bright cells | 0.010317646 | 0.730969539 |
| Tgd | -0.001337543 | 0.964448037 |
| Eosinophils | -0.035401476 | 0.237962588 |
| T helper cells | -0.057169608 | 0.056560856 |
| Tcm | -0.060282309 | 0.044358581 |
| Th17 cells | -0.073298933 | 0.014448442 |

Data were analyzed using the two-tailed statistical significance of Spearman's correlation coefficient.

**Supplementary Table S2.** Correlation of RNase1 expression with infiltrating immune cells for HNSCC

| **Immune cells** | **Correlation coefficient (R)** | ***P* value** |
| --- | --- | --- |
| Macrophages | 0.655206807 | 0 |
| DC | 0.534730326 | 0 |
| pDC | 0.493627624 | 2.58307E-32 |
| TReg | 0.4851913 | 4.01052E-31 |
| Cytotoxic cells | 0.477432904 | 0 |
| iDC | 0.470320088 | 0 |
| Th1 cells | 0.466613708 | 0 |
| T cells | 0.461131373 | 0 |
| Eosinophils | 0.425629251 | 0 |
| TFH | 0.424092784 | 0 |
| NK CD56dim cells | 0.38242361 | 0 |
| NK cells | 0.382057959 | 0 |
| Tem | 0.355805385 | 1.75694E-16 |
| CD8 T cells | 0.339566186 | 6.03852E-15 |
| aDC | 0.332357201 | 2.47699E-14 |
| Neutrophils | 0.311619674 | 1.07949E-12 |
| B cells | 0.289033081 | 4.59633E-11 |
| Mast cells | 0.224422536 | 3.83293E-07 |
| T helper cells | 0.223981992 | 4.04332E-07 |
| Th2 cells | 0.190773511 | 1.67816E-05 |
| NK CD56bright cells | 0.059050319 | 0.185591078 |
| Th17 cells | 0.028644821 | 0.520998485 |
| Tcm | -0.003974362 | 0.929055021 |
| Tgd | -0.122623336 | 0.005870287 |

Data were analyzed using the two-tailed statistical significance of Spearman's correlation coefficient.
